# Evaluating cell-specific gene expression using single-cell and single-nuclei RNA-sequencing data from human pancreatic islets of the same donors

**DOI:** 10.1038/s41598-025-21595-1

**Published:** 2025-10-16

**Authors:** Karin Engström, Åsa Nilsson, Jones K. Ofori, Nils Wierup, Karl Bacos, Charlotte Ling

**Affiliations:** 1 Epidemiology and Bioinformatics, Division of Occupational and Environmental Medicine, Department of Laboratory Medicine, Lund University, Lund, Sweden; 2 Human Tissue Lab, Department of Clinical Sciences in Malmö, Lund University Diabetes Centre, Lund University, Scania University Hospital, Malmö, Sweden; 3 Epigenetics and Diabetes Unit, Department of Clinical Sciences in Malmö, Lund University Diabetes Centre, Lund University, Scania University Hospital, Malmö, Sweden; 4 Neuroendocrine Cell Biology, Department of Experimental Medical Science, Lund University Diabetes Centre, Lund University, Scania University Hospital, Malmö, Sweden

**Keywords:** Single-nuclei RNA-sequencing, Single-cell RNA-sequencing, Pancreatic islets, Annotation, Marker genes, Type 2 diabetes, Biological techniques, Computational biology and bioinformatics, Genetics, Molecular biology

## Abstract

Single-cell and single-nuclei RNA-sequencing (scRNA-seq and snRNA-seq) analyze cell-specific transcriptomes. However, only snRNA-seq applies to frozen biobanked samples. For human pancreatic islets, marker genes and reference-based cell type annotation methods are mainly from scRNA-seq datasets and may not be suitable for snRNA-seq. We compared human islet scRNA-seq and snRNA-seq data from the same donors (N = 4) and evaluated annotation methods by studying cell type composition and gene detection, and identified novel marker genes. We compared cell type annotations: (1) manual annotation based on identified marker genes, (2) reference-based annotation using Azimuth’s scRNA-seq *pancreasref* dataset, or (3) Seurat’s label transfer from the Human Pancreas Analysis Program (HPAP) scRNA-seq dataset. ScRNA-seq and snRNA-seq identified the same cell types, but predicted cell type proportions differed. Cell type proportion-differences between annotation methods were larger for snRNA-seq. Reference-based annotations generated higher cell type prediction and mapping scores for scRNA-seq than snRNA-seq. Manual annotation identified the novel snRNA-seq markers *DOCK10, KIRREL3* (beta cells), *STK32B* (alpha cells), *MECOM*, *AC007368.1* (acinar cells), *LAMC2* and *SLC28A3* (ductal cells), which improve snRNA-seq-based annotation. We confirmed *ZNF385D* as a snRNA-seq beta cell marker and *ZNF385D* silencing reduced insulin secretion. In conclusion, this study discovered novel snRNA-seq cell type marker genes in human pancreatic islets, and highlights the need for tailored snRNA-seq annotation strategies.

**Supplementary Information:**

The online version contains supplementary material available at 10.1038/s41598-025-21595-1.

## Introduction

Type 2 diabetes (T2D), a disease characterized by chronic hyperglycemia, is increasing at an alarming rate worldwide. Glucose homeostasis is achieved by secretion of glucose-lowering insulin and glucose-elevating glucagon from pancreatic beta and alpha cells, respectively. In healthy individuals, insulin secretion is balanced against insulin sensitivity in peripheral tissues^1^. Somatostatin, secreted from pancreatic delta cells, also impacts glycemia by inhibiting insulin and glucagon release^2^. Other cell types in pancreatic islets that influence glucose levels include gamma cells, which produce pancreatic polypeptide, and epsilon cells, which produce ghrelin^3,4^. People with T2D are unable to maintain normoglycemia mainly due to insufficient insulin release from the beta cells^5^. To dissect the molecular mechanisms that underlie both hyperglycemia and normoglycemia in people with and without T2D, it is essential to study gene regulation in all islet cell types.

Earlier studies from our group and others used RNA-sequencing and microarray-based methods to identify alterations in the transcriptome and epigenome in whole human islets from donors with T2D versus non-diabetic controls^6–12^. Although many results in those studies have been technically and biologically replicated and functionally validated, they are limited in that they did not allow for cell type-specific analyses.

Single-cell RNA-sequencing (scRNA-seq) analyses gene expression of individual cells and can thus help us understand the pancreatic islet transcriptome at cell-specific resolution. Several studies have used scRNA-seq to investigate alterations in the transcriptome in human islet cells from donors with T2D versus non-diabetic controls^13–16^. However, the overlap in shared differentially expressed genes (DEGs) between these studies was modest^17–19^. This may be due to smaller sample sizes or to the inherent drawbacks of studying single cells, such as the potential for stress-induced transcriptional artifacts introduced by single-cell dissociation and the incompatibility of scRNA-seq with frozen archived material from biobanks, where most human islet samples are typically deposited. One solution is to perform single-nuclei RNA-sequencing (snRNA-seq), which can be done on frozen samples, allowing analysis of available larger biobanks^20,21^, and showing fewer technical issues due to cell dissociations. However, the transcripts analyzed by scRNA-seq and snRNA-seq are different: scRNA-seq analyses both nuclear and cytoplasmic transcripts, whereas snRNA-seq analyses primarily nuclear transcripts, leading to a bias towards nascent or incompletely spliced transcripts. A few studies have performed snRNA-seq in human pancreatic islets from donors with T2D versus non-diabetic controls^22,23^ and recent studies compared scRNA-seq and snRNA-seq in islets from one to three donors^24–26^. These studies found that snRNA-seq identified most human islet cell populations and thus could be a good alternative to scRNA-seq.

A significant challenge in scRNA/snRNA-seq studies is the correct annotation of cell types, which can be performed using, e.g., manual or reference-based approaches. Manual annotation is based on a library of marker genes, whose expression is higher in, and therefore characteristic of, a particular cell type. Reference-based annotation compares a generated expression profile to published reference datasets, where each individual cell or nucleus (barcode) has already been annotated with a predicted cell type. Inferred cell types are assigned to each barcode in a query dataset based on the most similar reference sample(s). This process depends on the reference data set’s quality and annotation; most marker genes and published reference data sets for human pancreatic islets are from scRNA-seq and not snRNA-seq studies^13–15,27–30^. Comparative studies have observed that snRNA‐seq better preserves the in situ molecular state, particularly for markers that are altered upon cell isolation^21^. The dissociation process for scRNA‐seq can introduce stress responses where scRNA‐seq might not fully recover specific cell populations; thus, these cell populations would not be found in snRNA-seq data when using a scRNA-seq dataset as a reference. This has frequently been seen among neuronal cell types, where some cell types are more vulnerable to dissociation^31^, but also in kidney and liver cells, where certain rare cell types identified by snRNA-seq were missing in scRNA-seq^32,33^. Thus, snRNA‐seq may yield more consistent cell-type profiles and better replicate the in vivo transcript distribution compared to scRNA‐seq. Kang et al.^25^ identified new snRNA-seq marker genes in human islets that may enable more accurate cell type annotation when analysing nuclear transcripts compared with the marker genes previously identified by scRNA-seq, indicating that marker gene selection and annotation should be performed using specific snRNA-seq cell-type markers for nuclei data.

**Fig. 1:**
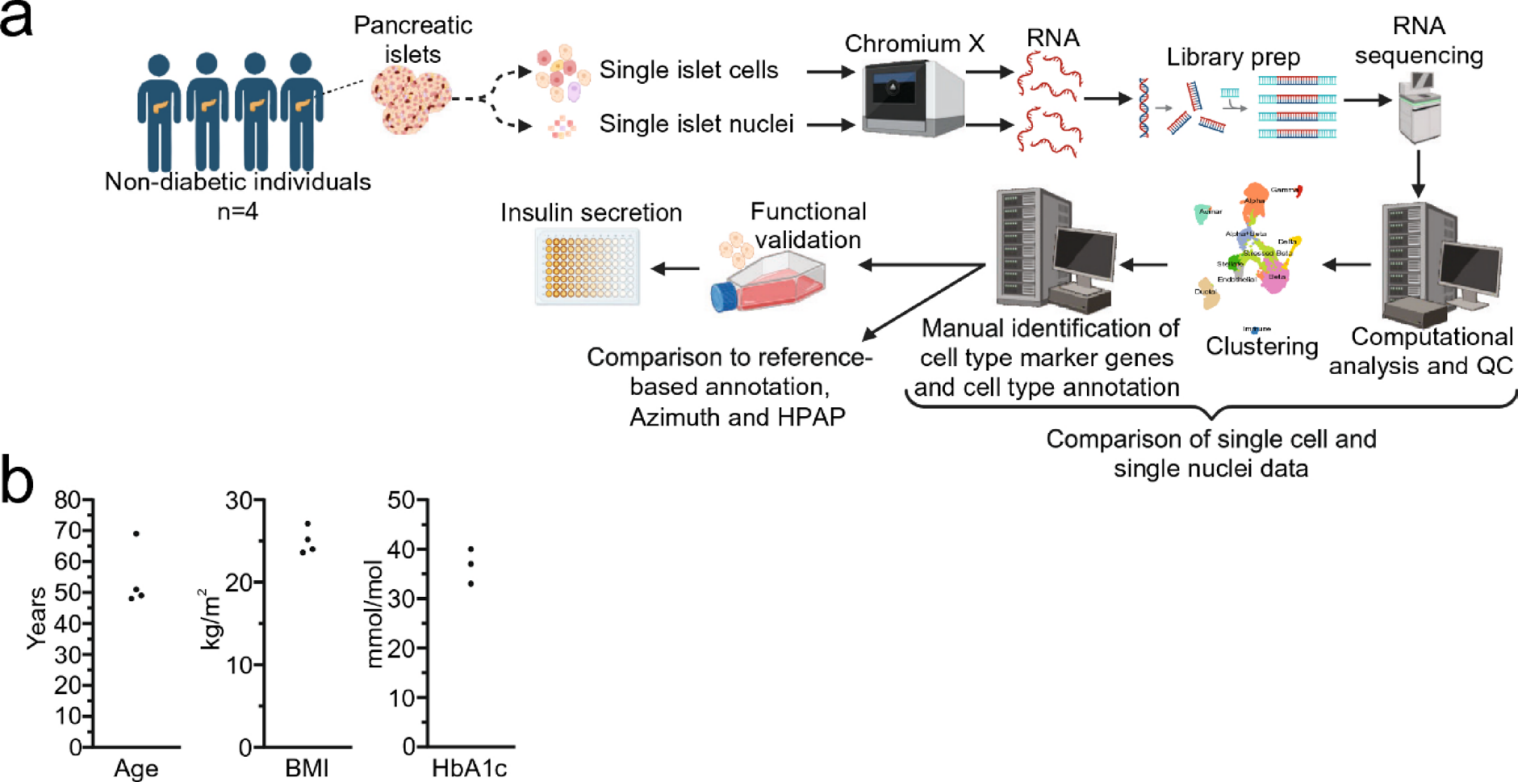
Study design and donor characteristics. (**a**) A schematic picture of the study design, including four non-diabetic donors, while (**b**) shows donor characteristics for age, body mass index (BMI), and HbA1c (a measure of average blood glucose levels over the past months, used to diagnose prediabetes and diabetes, one missing value).

Based on this, we aimed to compare scRNA-seq and snRNA-seq data generated from pancreatic islets of the same human donors (Fig. 1a). We compared manual annotation and two reference-based cell type annotation methods using scRNA-seq reference datasets^25,30^ on our scRNA-seq and snRNA-seq data. We then assessed differences in predicted cell type composition and gene detection between scRNA-seq and snRNA-seq. We also searched for potential novel snRNA-seq-specific marker genes for pancreatic islet cell populations that may be used to improve the annotation of cell types in snRNA-seq data. Finally, we studied insulin secretion after knockdown of the beta cell marker gene *ZNF385D* in INS-1 832/13 cells.

## Materials and methods

### Human pancreatic islets

Human pancreatic islets from four male non-diabetic multiorgan donors (Fig. 1b) were isolated at The Nordic Network for Islet Transplantation in Uppsala, Sweden, and were then sent to the Human Tissue Laboratory at Lund University Diabetes Center (LUDC). The islets were prepared by enzymatic digestion and density gradient separation, and islet preparation culture, purity, and count determinations were performed as described previously^34^. Islets were used fresh or after freezing at − 80 °C for scRNA-seq and snRNA-seq analysis, respectively.

### Dispersion of fresh islets into single cells

Freshly cultured (CMRL-1066 + 10% human serum) human islets were dissociated into single cells by Accutase (L0950; Biowest, USA). Briefly, 1000–2000 islet equivalents (IEQs)) were washed once in 5 ml Accutase. After removing the Accutase, 5 ml pre-warmed Accutase was added, and islets were incubated at 37 °C for 10 min with mixing every 2 min. Next, 5 ml of cold RPMI media was added, and a single-cell suspension was made by pipetting up and down. The suspension was passed through a 40 µm cell strainer and washed twice with PBS with 0.04% BSA (Merck, USA). Dead cells were removed using a Dead Cell Removal Kit (Miltenyi Biotec, Germany). A fraction of the cells was then stained with Trypan Blue and counted with a Bürker chamber.

### Single nuclei isolation from frozen islets

Single nuclei were isolated from frozen human islets using the Chromium Nuclei Isolation Kit (1000494; 10x Genomics, USA) with the whole process performed on ice and in 4 °C centrifuges. Briefly, frozen islets (1000–2000 IEQs) were transferred to a Dissociation Tube with cold Lysis Buffer (diluted 1:2 with PBS) and homogenized with a pestle. Additional diluted Lysis Buffer was added, and samples were incubated for 7 min on ice. The dissociated islets were transferred to a column assembled with a collection tube and centrifuged at 16,000*g* for 20 s. The flowthrough containing the nuclei was centrifuged for 3 min at 500*g*. The nuclei pellet was then resuspended in Debris Removal Buffer and centrifuged at 700*g* for 10 min. The supernatant was discarded, and the nuclei were washed twice in Wash Buffer and passed through a 40 µm cell strainer. The nuclei were centrifuged at 500*g* for 5 min, and the pellet was resuspended in Resuspension Buffer. A fraction of the nuclei was mixed with AO/PI (DeNovix, USA) at a 1:1 ratio and counted with the CellDrop Automated Cell Counter (DeNovix, USA).

### Generation of single-cell and single-nuclei RNA-sequencing data

Cells and nuclei were prepared following Chromium Next GEM Single Cell 3ʹ Reagent Kit v3.1 protocols and Chromium Next GEM Single Cell Multiome ATAC + Gene Expression Kit (10x Genomics, USA), respectively. Briefly, 9000–16,000 cells or nuclei were loaded into the Chromium Controller to generate Gel Beads-in-Emulsion (GEMs) with barcoded gel beads (one unique barcode is used for each cell/nucleus), a master mix, and partitioning oil on a chromium chip. A barcode is a unique nucleotide sequence assigned to each cell or nucleus during sample preparation, and the word barcode is used when referring to a cell or nucleus. Following GEM formation, cDNA with a 16nt 10x barcode and a 12nt Unique molecular identifier (UMI) was produced with reverse transcription. The cDNA was purified using Dynabeads and amplified via PCR.

Quality control and cDNA quantification were performed using High Sensitivity D5000 ScreenTapes (Agilent, USA). The library construction was then carried out, including fragmentation, adapter ligation, and sample index PCR. Final quality control and quantification of the libraries were performed using High Sensitivity D5000 ScreenTapes (Agilent, USA) and the Qubit dsDNA HS Assay Kit (Thermo Fisher Scientific, USA). The libraries were sequenced by the NovaSeq 6000 system (Illumina, USA) at the Center for Translational Genomics (CTG), Lund, Sweden.

### Preprocessing of sequencing data

The raw sequencing data were processed using the 10x Genomics Cellranger v.7.1.0 pipeline. Introns were included for both scRNA-seq and snRNA-seq. The GRCh38-2020-A library was used. Automatic cell calling was done in Cellranger, automatically or, when appropriate, via manual inspection of the inflection point in the barcode-rank plot.

### Quality control, ambient RNA correction, and doublet removal

Each sample was analyzed separately until after ambient correction, doublet removal, quality control (QC), and filtering away of barcodes with low quality. The R package SoupX^35^ was used to estimate and remove ambient RNA contamination. The SoupX corrected feature-barcode matrices were further processed using the R package Seurat (v5.1.0)^36^, where features present in fewer than 10 barcodes per sample and barcodes with fewer than 200 features were removed as an initial step. A feature is a measurable entity in the dataset, most commonly nuclear transcripts, but it also includes mitochondrial transcripts and ribosomal RNA. Predicted doublets were removed using the R package scDBlfinder^37^. Low-quality barcodes were further filtered away if they met any of the following criteria: fewer than 500 features detected in each cell/nucleus, fewer than 500 molecules (counts) detected within a cell/nucleus, a log10Genes per Unique Molecular Identifier (UMI) below 0.80 (denoting the complexity), a fraction of mitochondrial genes above 5%, or a fraction of ribosomal genes above 35%. To avoid modality (snRNA-seq or scRNA-seq)-specific bias, identical cut-offs were applied to both scRNA-seq and snRNA-seq datasets. The nuclear lncRNA gene metastasis-associated lung adenocarcinoma transcript 1 (*MALAT1)* can dominate sequencing reads, introduce bias, and potentially obscure the detection of less abundant transcripts. In studies where *MALAT1* is not of primary interest it can therefore be wise to remove it from the dataset before further analysis^38^. Thus, we removed *MALAT1* transcripts from the dataset. The function Seurat LogNormalize was then used for normalization.

### Integration and unsupervised clustering

We selected a subset of 2000 features that exhibit high barcode-to-barcode variation (i.e., highly expressed in some barcodes and lowly expressed in others), and the individual datasets were then integrated using Seurat Canonical Correlation Analysis (CCA)^36^. Focusing on these highly variable features in downstream analysis helps to highlight biologically relevant signals in single-cell/nuclei datasets^39^. The integrated dataset was further processed by scaling the data and regressing the number of features and fractions of mitochondrial genes. Principal component analysis (PCA) was then performed. The FindClusters function in Seurat, a Louvain graph-based approach, using the top 30 principal components with the resolution parameter set to 1, was used to identify distinct cell populations within our dataset, where the barcodes were clustered based on their transcriptomic profiles. Clusters were further evaluated for technical variables, including mitochondrial content, cells representing a single donor, extreme number of features, and high doublet score, and low-quality clusters were removed before re-clustering of the data.

### Cell type annotation

To annotate cell types, we compared three different approaches as described in more detail below: (1) manual annotation of clusters based on marker genes identified using Seurat’s FindAllMarkers function, (2) reference-based annotation using Azimuth with Azimuth’s scRNA-seq *pancreasref* dataset^36^, and (3) reference-based annotation with Seurat’s label transfer using CCA integration with the Human Pancreas Analysis Program (HPAP) non-diabetic scRNA-seq dataset^30^. To assess the agreement between these three annotation methods, we compared the distribution of predicted cell types and visualized the results using Uniform Manifold Approximation and Projection (UMAP). Additionally, for the reference-based annotations, we calculated median prediction scores (both) and mapping scores (only available for Azimuth) across all barcodes and per cell type.

(1) Manual annotation: We first performed a marker gene analysis to identify cluster-specific differentially expressed genes (DEGs). Using the FindAllMarkers function in Seurat, we applied the Wilcoxon rank-sum test to compare each cluster against all others, separately for the snRNA-seq and scRNA-seq datasets. Genes were classified as marker genes if they exhibited a log₂ fold change (FC) > 1 in one cluster compared to the cells of all other clusters and were expressed in at least 25% of barcodes within a given cluster and false discovery rate (FDR) < 0.05. Cell types were then assigned cluster-wise by comparing our identified cluster-specific marker genes with canonical markers for pancreatic cell types reported in the literature^14,25,27,29^: *INS* (beta cells); glucagon* (GCG)* (alpha cells); *SST* (delta cells); *PPY* (gamma cells); *PRSS1* and *REG1A* (acinar cells); *KRT19* (ductal cells); *PTPRC* (immune cells); *PECAM1* (endothelial cells); *COL1A1* (activated stellate cells); *PRKG1* (quiescent stellate cells); and *SOX10* and *S100B* (Schwann cells). We also used marker genes identified by Kang et al.^25^ in snRNA-seq data; *ZNF385D, TRPM3, LRFN2*, and *PLUT* for beta cells; *PTPRT, FAP, PDK4*, and *LOXL4* for alpha cells; *LRFN5, ADARB2, ERBB4*, and *KCNT2* for delta cells; *CACNA2D3, THSD7A, CNTNAP5*, and *RBFOX3* for gamma cells. (2-3) Azimuth and HPAP reference-based annotation: The publicly available Azimuth Human Pancreas reference (*pancreasref*) comprises six scRNA-seq pancreas datasets^13–15,27–29^ integrated into a single reference by Seurat’s integration framework. Our query dataset was mapped to *pancreasref* using the Azimuth R package (version 0.4.6) and the core function RunAzimuth, following the workflow outlined in the official tutorial (https://satijalab.github.io/azimuth/articles/run_azimuth_tutorial.html). This pipeline included dimensionality reduction and automated cell type annotation, and utilized the unnormalized expression data in the ‘counts’ slot of the ‘RNA’ assay. The HPAP dataset^30^ contains scRNA-seq profiles from non-diabetic donors and is publicly available at http://www.gaultonlab.org/pages/Islet_expression_HPAP.html. Seurat’s FindTransferAnchors() and TransferData() functions were used for annotation, enabling label transfer from the reference to our dataset. For both references, cell identities were transferred onto our query based on anchor correspondence in a shared low-dimensional space. In the case of Azimuth, SCTransform normalization was applied internally to harmonize the query data with the SCTransformed reference. For the HPAP reference, both query and reference datasets were normalized using LogNormalize before integration, and anchors were identified using CCA. Anchors are pairs of cells across datasets that are identified as biologically similar based on the transcriptome, forming “guideposts” for how to align the reference and query datasets.

The pair-wise overlaps between the annotated cell types were visualized by bar plots, and fractions annotated to the same cell type when comparing two annotation methods were calculated to compare these three annotations. We examined the prediction scores (Azimuth and HPAP) to assess annotation confidence and compared them between annotation methods and scRNA-seq and snRNA-seq. Cell prediction scores range from 0 to 1 and reflect the confidence associated with each annotation, and a high prediction score reflects predictions supported by multiple consistent anchors. For Azimuth, we also examined mapping scores. The mapping score ranges from 0 to 1 and reflects the confidence that a barcode is well represented by the reference and how well the unique structure of a cell’s local neighbourhood is preserved during reference mapping. To assess the consistency between cell type annotation methods, we calculated the Jaccard similarity index for each pair of annotation methods. First, cell type labels were harmonized to ensure comparability across methods:

1. ductal and *MUC5B*^+^ ductal (HPAP) were combined as ductal,
2. gamma + epsilon (HPAP) was compared to gamma for azimuth and manual annotation,
3. mast and macrophage (Azimuth) were combined as immune, and
4. activated stellate, quiescent stellate, and Schwann were combined as mesenchymal.

However, the alpha + beta category was not collapsed. We are aware that this can influence the values for comparisons with Azimuth annotation, since Azimuth does not have an alpha + beta category as manual and HPAP annotation do. For each cell type, we calculated the Jaccard index as the number of cells assigned to that cell type by both annotation methods in a pair-wise comparison (intersection) divided by the number of cells assigned to that cell type by at least one of the methods (union). Values range from 0 (no overlap) to 1 (perfect agreement). Cell type–specific Jaccard scores were then averaged to obtain an overall similarity matrix between annotation methods. To assess annotation consistency between scRNA-seq and snRNA-seq, we calculated weighted Jaccard indices per donor and annotation method, which accounts for both shared cell types and their relative proportions. In addition, we used paired Wilcoxon signed-rank test (further described in the methods section) to 1) assess whether the predicted cell type proportions differed between scRNA-seq and snRNA-seq within the three annotation approaches, and 2) whether cell type proportions differed between annotation methods within the same modality.

### Identification of more robust cell type-specific marker genes

To identify novel cell type-specific marker genes that characterize each cell type and are low-frequent in other cell types, we conducted a second FindAllMarkers analysis by applying stricter criteria and comparing the annotated cell types derived from the manual annotation of the original clusters. Here, we required marker genes to have a log₂ FC > 1 and to be expressed in at least 50% of barcodes within a given cell type. To identify genes that are differently expressed between scRNA-seq and snRNA-seq in the same cell type, we conducted a FindAllMarkers analysis. We considered genes with a log₂ FC > 1, expressed in at least 25% of barcodes within a given cell type, and with FDR < 0.05 to be differently expressed between scRNA-seq and snRNA-seq.

### Comparison of genes expressed in scRNA-seq and snRNA-seq

A gene was considered expressed if it had a UMI count ≥ 3 for at least one barcode. We evaluated the number of genes expressed in at least one barcode, as well as in at least 10% of the barcodes, and the overlap in expressed features between the scRNA-seq and snRNA-seq data. We evaluated the number of features expressed per cell type (based on cell type prediction from manual annotation) and how the expression per cell type overlapped and differed between cells and nuclei. To compare the marker gene detection rate (defined as the fraction of cells a gene is considered expressed in) between scRNA-seq and snRNA-seq for the same donor, we performed paired statistical analyses as further described in the statistics section.

### siRNA-mediated knockdown and insulin secretion

The rat beta cell line 832/13 INS1^40^, a kind gift from Professor Christopher Newgard (Duke University, Durham, NC, USA), was transfected with 25 nM negative control siRNA (Silencer Select Negative Control No. 2 siRNA, Thermo Fisher Scientific), or one of two siRNAs targeting *Zfp385d*, the rat homologue of human *ZNF385D* (s156987 and s156988, Thermo Fisher Scientific), by using Lipofectamine RNAiMAX (Thermo Fisher Scientific). RNA was isolated 72 h after transfection with miRNeasy (Qiagen, Hilden, Germany) and converted to cDNA with RevertAid cDNA synthesis kit (Thermo Fisher Scientific). Knockdown was analysed by qPCR with TaqMan assays (Thermo Fisher Scientific) for *Zfp385d* (assay id Rn01772858_m1) and *Hprt1* (Rn01527840_m1), and *Ppia* (Rn00690933_m1, data not shown) as endogenous controls. Quantification was done with the ΔΔCt method. For secretion experiments, transfected cells were washed once in secretion assay buffer (SAB: 114 mM NaCl, 4.7 mM KCl, 1.2 mM KH_2_PO_4_, 1.16 mM MgSO_4_, 20 mM HEPES, 2.5 mM CaCl_2_, 25.5 mM NaHCO_3_, 0.2% bovine serum albumin, pH 7.2) containing 2.8 mM glucose and then preincubated for 2 h at 37 °C in the same buffer. Insulin secretion was then stimulated for 1 h with SAB containing either 2.8 or 16.7 mM glucose. Secreted insulin was measured with High range rat insulin ELISA (Mercodia, Uppsala, Sweden) and normalised to total protein as determined by BCA assay (Themo Fisher Scientific).

### Statistics

Summary data are presented as median (quartile 1; quartile 3) unless stated otherwise. We compared QC metrics, cell-type proportions, and marker gene detection between snRNA-seq and scRNA-seq on a per-donor basis by using the paired Wilcoxon signed-rank test. All tests were two-sided unless otherwise stated. We first aggregated per-cell measurements (such as percent fraction of mitochondrial genes) as per-donor average for snRNA-seq and scRNA-seq separately, to avoid pseudo-replication. Effect sizes and confidence intervals are reported alongside p-values to account for the limited statistical power inherent to the sample size (n = 4 paired donors). Effect sizes are shown as Hodges–Lehmann (HL) location shift (the median of paired differences (snRNA-seq–scRNA-seq)). Thus, negative estimates indicate lower values in snRNA-seq relative to scRNA-seq, and vice versa. To provide stable uncertainty quantification with small n, 95% confidence intervals for the HL shift were obtained via nonparametric bootstrap (percentile method; 10,000 resamples). Where an exact Wilcoxon confidence level was not achievable, the bootstrap CI was reported. Multiple p-values were adjusted using the Benjamini–Hochberg method, applied separately within each analysis. All statistical analyses were implemented in R (version 4.3) using the dplyr and stats packages.

## Results

### RNA-seq analysis of single cells and nuclei from human pancreatic islets

Fresh and frozen pancreatic islets from four male donors were used for scRNA-seq and snRNA-seq profiling, respectively. The study design and the characteristics of the islet donors are presented in Fig. 1a,b. Descriptive quality metrics and barcode summary data per sample are shown in Table 1 and Supplementary Fig. 1. The median ambient RNA fraction per sample was estimated to be 1.9% (range 1–2.4) for cells and 3.2% (range 1.9–7.4) for nuclei (HL shift = 1.3%, 95% CI 0.8–5.0, FDR = 0.12, when comparing cells and nuclei), with *INS* (encoding insulin) being the most abundant transcript (median *INS* of the total ambient RNA in cell and nuclei samples were 28 and 27%, respectively). The median doublet fraction was estimated to be 9.5% (range 3.2–11) for cells and 7.6% (range 6.6–14) for nuclei (HL shift = 0.6%, 95% CI − 3.4 to 4.5, FDR = 0.58). The median fraction of intronic reads per sample was 19% (range 14–22) for cells and 54% (range 50–56) for nuclei (HL shift = 35%, 95% C.I 33.4–36.6, FDR = 0.12).

**Table 1:** Overview of quality metrics and barcode statistics of scRNA-seq and snRNA-seq data.

Sample	Type	Number of barcodes	Mean reads per barcode^a^	Median genes per barcode^a^	Intronic reads (%)^a^	Ambient RNA (%)^a^	Doublets (%)^a^	Mitochondrial gene fraction^a^	Ribosomal gene fraction^a^	Counts per barcode^b^	Features per barcode^b^	Complexity (log 10 features per barcode/log 10 counts per barcode)	Number of barcodes included in downstream analyses^c^
Donor 1	Cells	7500^d^	56262	4779	19.0	2	11	3.7 (2.5, 5.6)	6.9 (5.5, 9.0)	17,820 (3993, 32,829)	4508 (1567, 6081)	0.86 (0.83, 0.89)	4241
Donor 2	Cells	6999	71058	4542	21.9	2.4	9.1	3.5 (0.94, 7.0)	11 (7.1, 15)	15,829 (2660, 35,147)	4132 (1028, 6591)	0.86 (0.83, 0.90)	3020
Donor 3	Cells	2133	43,747	6121	19.9	1	3.2	3.5 (0.53, 5.5)	11 (7.8, 15.8)	2989 (1889, 96,271)	5946 (790, 9558)	0.83 (0.80, 0.89)	1003
Donor 4	Cells	6800^d^	110,065	4578	14.3	1.9	10	2.8 (1.6, 5.7)	8.6 (3.5, 11.4)	19,469 (5480, 3721)	4219 (1934, 6133)	0.85 (0.82, 0.88)	3607
Donor 1	Nuclei	4974	51,841	1831	55.6	3.6	7.6	< 0.001 (< 0.001, 0.084)	0.44 (0.31, 0.66)	3691 (2712, 4977)	1740 (14,515, 2132)	0.91 (0.90, 0.92)	4273
Donor 2	Nuclei	9720	36,703	2238	55.3	7.4	14	< 0.001 (< 0.001, 0.084)	0.19 (0.11, 0.32)	4248 (2989, 5651)	1991 (1570, 2414)	0.91 (0.90, 0.92)	7783
Donor 3	Nuclei	5309	63,819	2644	53.4	1.9	7.7	0.007 (< 0.001, 0.12)	0.27 (0.19, 0.37)	6120 (3744, 9132)	2506 (1824, 3175)	0.90 (0.88, 0.92)	4683
Donor 4	Nuclei	4307	146,166	2746	50.7	2.7	6.6	0.014 (< 0.001, 0.24)	0.33 (0.24, 0.54)	6634 (5024, 8511)	2657 (2192, 3110)	0.89 (0.88, 0.90)	3545

^a^The median, quartile 1, and quartile 3 are presented. For the metrics derived from Cellranger web summaries, only the mean (reads per barcode) and median (genes per barcode and intronic reads) are presented. For ambient contamination and doublet fraction, only the mean is presented.

^b^After removing features present in less than 10 barcodes, barcodes with less than 200 features, and barcodes flagged as doublets.

^c^After removal of barcodes as stated in ^b^, as well as filtering based on removing those that met one or many of the following criteria: less than 500 features, less than 500 counts, log10GenesPerUMI below 0.80, fraction of mitochondrial genes above 5%, and fraction of ribosomal genes above 35%

^d^Cell calling for these samples was made with manual inspection, where the—force-cells command with a value of called cells based on manual inspection of the inflection point in the barcode-rank plot.

After pooling cells and nuclei from the four donors, the initial datasets from Cellranger included 23,432 cells and 24,310 nuclei. In the pooled data, the median number of features (i.e., transcripts) was 4361 per cell (quartile 1[Q1]: 1434; quartile 3[Q3]: 6428) and 2120 (Q1: 1613; Q3: 2746) per nucleus (HL shift = 2478, 95% C.I 1562–3440, FDR = 0.12) The fractions of mitochondrial encoded genes and ribosomal RNAs per sample were, as expected, very low in nuclei (median < 0.001%, Q1: < 0.001; Q3: 0.048, and median 0.18%, Q1: 0.28; Q3: 0.45, respectively), while it was higher in cells (median 3.4%, Q1: 1.6; Q3: 5.9, and median 8.6%, Q1; 5.8; Q3: 13, respectively), HL shift = 3.5%, 95% C.I 2.8–3.7, FDR = 0.12 for fractions of mitochondrial genes, and HL shift = 9.1%, CI 6.5–10.9, FDR = 0.12 for fractions of ribosomal genes. Applying the same cut-offs for mitochondrial and ribosomal transcript fractions in scRNA-seq and snRNA-seq did not impact the results, as nuclei contained very low levels of these transcripts.

After ambient correction, doublet removal, and filtering, 11,871 cells and 20,284 nuclei remained, which were included in the downstream analysis. The lower number of retained cells vs. nuclei after QC was partly explained by low-quality cells (7045 cells) filtered away due to high mitochondrial RNA gene content. The corresponding number of filtered nuclei was 1084. Additionally, more cells than nuclei were filtered away due to the cutoffs for number of features (1270 cells; 570 nuclei) and complexity (2507 cells; 163 nuclei).

### Unsupervised clustering to identify distinct cell populations

**Fig. 2:**
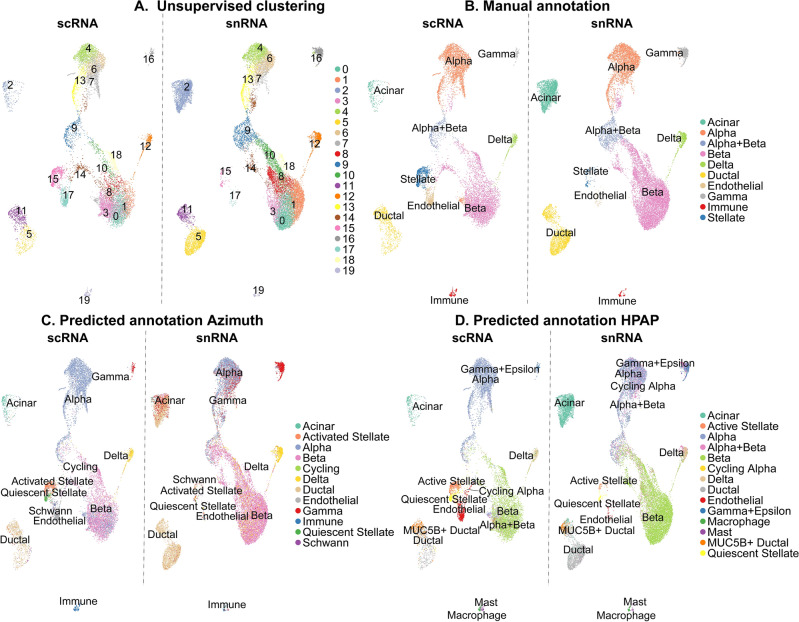
Uniform Manifold Approximation and Projection (UMAP) showing cell types identified through different annotation methods. The panels show (**a**) clusters from unsupervised clustering, (**b**) manually annotated cell types, (**c**) annotation according to Azimuth and its *pancreasref*, and (**d**) annotation according to label transfer using CCA integration with scRNA-seq data from Human Pancreas Analysis Program (HPAP) as reference. The figures are split by scRNA-seq (left) and snRNA-seq (right).

To identify distinct cell populations, we integrated the scRNA-seq and snRNA-seq datasets and performed unsupervised Louvain clustering, initially identifying 24 clusters. We then assessed the quality control metrics for these 24 clusters, including detected features per cell/nucleus, UMI counts, and mitochondrial and ribosomal gene percentages. Subsequently, four clusters were removed due to the following technical artifacts: (1) consisted mainly of nuclei with high mitochondrial content (mean 2.9% vs. 0.40% for all nuclei), (2) contained only cells from a single donor, (3) included mainly nuclei with an exceptionally high number of features (mean 5771 vs. 2223 for all nuclei), likely representing doublets missed by ScDblFinder (doublet score: 0.26 vs. 0.090 for all nuclei), and (4) consisted of mainly low-complexity cells with fewer features (mean 1731 vs. 4701 for all cells) and UMI counts (mean 6698 vs. 20,752 for all cells). After removing these clusters, re-clustering yielded 20 clusters (Fig. 2a) containing 30,954 barcodes (11,207 cells and 19,747 nuclei). The distribution of cells and nuclei in the UMAP and the clusters is shown in Supplementary Fig. 2.

### Cell type annotation

To annotate the cell types for the clusters presented in Fig. 2a, we used three different approaches: manual annotation based on known marker genes (Fig. 2b), and reference-based annotation using Azimuth and the *pancreasref* dataset (Fig. 2c)^36^, or cell type label transfer with CCA integration in Seurat with the HPAP non-diabetic dataset^30^ (Fig. 2d), as reference.

The manual annotation method identified clusters corresponding to the endocrine cell types alpha, beta, delta, and gamma, a cluster with a mixture of alpha and beta cells, as well as acinar, ductal, and non-parenchymal endothelial, immune, and stellate cells (Fig. 2b). Four clusters were annotated as alpha cells (clusters 4, 6, 7, and 13), and seven as beta cells (clusters 0, 1, 3, 8, 10, 14, and 18) (Fig. 2a,b). One intermediate cluster (cluster 9) was annotated as alpha + beta cells, as high expression of both *INS* and *GCG* was evident in the cluster. Beta cell clusters closer to the alpha clusters in the UMAP (clusters 1, 14, and 18) showed fewer beta cell-specific marker genes^25^ and were enriched for stress-related genes, including heat shock proteins (HSPs), suggesting that these clusters may represent stressed, dying, or transitioning beta cells. These cells were labelled “stressed beta cells” (Supplementary Table 1). For the four alpha cell clusters identified, marker genes were broadly consistent across clusters (Supplementary Table 1).

Both reference-based annotations identified all cell types from the reference datasets in our scRNA-seq and snRNA-seq datasets, except for epsilon cells present in the Azimuth reference dataset, pancreasref, but not in our dataset. For Azimuth, the cell types identified were the endocrine cell types alpha, beta, delta, and gamma; the exocrine cell types acinar and ductal; and non-parenchymal cell types endothelial, immune, activated stellate, quiescent stellate, and Schwann, as well as cycling cells (the latter only present in scRNA-seq) (Fig. 2c).

For HPAP, the cell types identified were alpha, cycling alpha, alpha + beta, beta, delta, gamma + epsilon, ductal, MUC5B^+^ ductal, endothelial, macrophage, mast, activated stellate, quiescent stellate, Schwann, as well as cycling cells (Fig. 2d).

### Comparison of predicted cell type composition between snRNA-seq and scRNA-seq

A comparison of the predicted cell type composition between the snRNA-seq and scRNA-seq data is shown in Table 2 and Supplementary Fig. 3. The predicted beta cell fractions were consistently higher in snRNA-seq (40–44%, depending on annotation method) than in scRNA-seq (29–40%, FDR=0.62 for manual annotation, 0.14 for Azimuth, and 0.21 for HPAP), while the predicted alpha cell fractions were consistently lower in nuclei (17–19%) than in cells (29–44%, FDR=0.62 for manual annotation, 0.14 for Azimuth, and 0.21 for HPAP). For all annotation methods, the predicted fractions of immune, endothelial, and stellate cell types were higher in cells (4.6–7.6%) than in nuclei (0.1–0.6%, FDR=0.14–0.21, depending on annotation method). For the manual annotation and HPAP, the predicted fractions of acinar cells were also substantially higher in nuclei (12.3–13.3%) than in cells (2.3–2.6%, FDR = 0.21), while the opposite was seen for Azimuth (0.3% in nuclei and 1.9% in cells, FDR = 0.14). We quantified the concordance of predicted cell type composition for scRNA-seq and snRNA-seq, per annotation method, by using the weighted Jaccard index. The manual annotation showed the highest concordance between scRNA-seq and snRNA-seq (Supplementary Fig. 3), although no pairwise comparisons were statistically significant (all had FDR = 0.38).

**Table 2:** Percentages of cell types annotated using manual or reference-based (Azimuth and HPAP) annotation.

Predicted Celltype^a^	Dataset (scRNA/snRNA) and annotation method					
	*scRNA,*	*snRNA,*	*scRNA,*	*snRNA,*	*scRNA,*	*snRNA,*
	*Manual annotation*	*Manual annotation*	*Azimuth*	*Azimuth*	*HPAP*	*HPAP*
Acinar	2.30	12.00	1.90	0.30	2.60	13.00
Alpha	29.00	19.00	44.00	17.00	35.00	18.00
Alpha + Beta	3.60	4.70			3.80	11.00
Beta (of which % stressed beta cells)	40 (8.6)	44 (9.1)	29.00	40.00	33.00	42.00
Cycling	NA	NA	0.02		0.03	0.03
Delta	3.00	3.70	3.10	8.80	2.50	2.20
Ductal (incl MUC5B^+^ Ductal)	7.60	11.00	7.40	19	7.10	10
Endothelial	5.10	0.20	4.60	0.10	6.10	0.30
Gamma (Gamma + Epsilon for HPAP)	0.80	3.70	1.50	14.10	0.70	1.60
Immune total	1.50	0.60	2.10	0.40	1.50	0.70
Macrophage	NA	NA			0.60	0.40
Mast	NA	NA			0.90	0.30
Mesenchymal cells total	6.90	0.60	6.80	0.10	7.60	0.60
Schwann	NA	NA	0.50	0.02	NA	NA
Activated_stellate	NA	NA	4.50	0.10	4.70	0.30
Quiescent_stellate	NA	NA	1.80		2.90	0.30

^a^In HPAP, there was also a “cycling alpha” cell type. However, these were very few and were not included in the summary.

^b^NA = not available using the current annotation method.

### Comparison of cell type predictions for the different annotation methods

Next, we compared the predicted cell type composition between the three annotation methods and found that cell type fractions differed (Table 2, Supplementary Fig. 4 and Supplementary Table 2). Although we collapsed the cell types to be as comparable as possible between the annotation methods, some annotation differences were influenced by differences in cell type annotations in the reference datasets, e.g., while HPAP included an alpha + beta cell type, Azimuth did not.

For scRNA-seq, the fractions of predicted cell types were relatively consistent between the annotation methods. However, we found larger differences in predicted cell type fractions between annotation methods for the snRNA-seq data, for example, Azimuth annotated a relatively large fraction of nuclei as delta (8.8%) or gamma (14%), and these predicted delta or gamma nuclei were present among nuclei predicted as several other cell types in the other annotation methods (Supplementary Fig. 4). These fractions were substantially higher than for the manual annotation and HPAP, where the fractions of delta and gamma cells in the snRNA-seq data, were predicted to be 3.7% (FDR = 0.16 compared to Azimuth) and 3.7% (FDR = 0.16), respectively, for the manual annotation, and 2.2% and 1.6%), respectively, for HPAP (FDR = 0.16 for both comparisons). Additionally, Azimuth classified a substantially smaller fraction of nuclei as acinar (0.3%) compared to manual annotation and HPAP (12%, FDR = 0.16, and 13%, FDR = 0.16), while a larger fraction was predicted to be ductal (19%) compared to manual annotation and HPAP (11%, FDR = 0.16, and 10%, FDR = 0.16). For scRNA-seq, the correspondence between pair-wise predicted cell types for the different annotation methods was high (Supplementary Fig. 4), and pair-wise comparisons showed that over 75% of the cell type predictions could be replicated when using another annotation method (excluding the alpha + beta cell type) (Table 3). However, for some pair-wise comparisons, the overlap fraction between predicted gamma cells was lower than for the other cell types (< 75% for four out of six comparisons). For snRNA-seq, the overlap was very low for acinar cells in the pair-wise comparisons where the Azimuth annotation was one of the annotations compared (Table 3, Supplementary Fig. 4). Four cell types (acinar, alpha + beta, gamma, and endothelial) showed an overlap below 50% among at least two pair-wise comparisons for snRNA-seq. In total, when excluding the alpha + beta cell type, one pair-wise comparison (gamma, HPAP/Azimuth) showed an overlap below 50% in scRNA-seq (Table 3, Supplementary Fig. 4), while eleven pair-wise comparisons showed an overlap below < 50% in snRNA-seq. We also performed Jaccard similarity analyses between annotation methods and for scRNA-seq this revealed a good concordance between annotation approaches in the pair-wise comparisons (manual vs. Azimuth = 0.81, manual vs. HPAP = 0.85, HPAP vs. Azimuth = 0.84). For snRNA-seq, the concordance was slightly lower (manual vs. Azimuth = 0.70, manual vs. HPAP = 0.83, HPAP vs. Azimuth = 0.63).

**Table 3:**
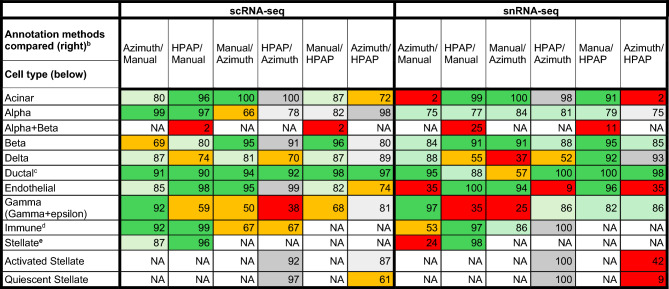
Pair-wise comparison table showing similarity of cell type annotations between two methods, expressed as percentage.

^a^The cells are coded according to the fraction overlap. Dark green>90%, bright green=75–90%, Orange=50–75%, Red<50%

^b^The pair-wise comparison values indicate the percentage of barcodes annotated as a given cell type by one method that is also annotated as the same cell type by the comparison method. For example, in the HPAP/Manual column, the percentage reflects the proportion of cells labeled as a specific cell type in the manual annotation that are also labeled the same in the HPAP annotation. If 260 cells are annotated as acinar in the manual annotation, and 250 of those are also labeled as acinar in HPAP, the overlap is calculated as (250/260)×100=96%. Conversely, the Manual–HPAP column reflects the proportion of cells labeled as a specific type in the HPAP annotation that are also labeled as such in the manual annotation. If 287 cells are annotated as acinar in HPAP, and 250 of those are also annotated as acinar manually, the overlap is (250/287)×100=87%. Percentages are, therefore, not necessarily symmetric between methods.

^c^The HPAP Ductal and MUC5B+Ductal cell types are merged into a Ductal cell type.

^d^The HPAP Macrophage and Mast cell types are merged into an Immune cell type.

^e^The Activated and Quiescent stellate cell types in Azimuth and HPAP are merged as a Stellate cell type when compared to the manual annotation.

### Cell type prediction and mapping scores for reference-based annotations

We proceeded by evaluating annotation confidence and reference mapping quality. Prediction scores assess the confidence in the cell type label assigned to each barcode, based on similarity to the reference. Mapping scores assess the confidence in how well a query barcode’s (i.e., barcodes from our scRNA-seq or snRNA-seq dataset) overall transcriptome aligns with the reference embedding. Cell type prediction scores (0–1) were calculated for both reference-based annotation methods, while mapping scores (0–1) were only calculated for Azimuth (this was not available for HPAP). These metrics differed between scRNA-seq and snRNA-seq, and between cell types and annotation methods (Supplementary Tables 3 and 4). The prediction scores were relatively high for scRNA-seq (median: 1, Q1: 0.92; Q3: 1 for Azimuth and median 1, Q1: 0.88; Q3: 1 for HPAP), while the scores were lower for snRNA-seq (median 0.67, Q1: 0.48; Q3: 0.88 for Azimuth and median 0.90, Q1: 0.73; Q3: 0.98 for HPAP). Additionally, for the scRNA-seq data, Azimuth generated median prediction scores above 0.9 for seven of twelve cell types (acinar, alpha, beta, delta, ductal, endothelial, and immune cells), while HPAP had prediction scores above 0.9 for eight of fourteen cell types (acinar, activated stellate, alpha, beta, endothelial, macrophage, mast, and quiescent stellate cells). However, for snRNA-seq, only one of the cell types had a prediction score above 0.9 for Azimuth (ductal cells) while six cell types had a prediction score above 0.9 for HPAP (acinar, activated stellate, beta, cycling alpha, ductal, and quiescent stellate cells), respectively. The Azimuth mapping scores also differed between scRNA-seq and snRNA-seq (Supplementary Table 4). While the median mapping scores were relatively high for scRNA-seq, (median: 0.79, Q1: 0.66, Q3: 0.89), they were considerably lower for snRNA-seq, (median 0.27, Q1: 0.16, Q3: 0.40). For snRNA-seq, beta cells had the lowest mapping score (median 0.15, Q1: 0.11, Q3: 0.25).

Together, these data highlight that the reference-based annotations using scRNA-seq references were suitable for scRNA-seq data, but not for snRNA-seq data. HPAP had higher prediction scores than Azimuth for more cell types when annotating snRNA-seq data.

### Comparison of detected genes in scRNA-seq and snRNA-seq data

A total of 23,149 genes were detected using either scRNA-seq or snRNA-seq at a threshold of UMI ≥ 3, with 18,649 genes (81%) identified using both methods. 3529 genes (15%) were exclusively detected in scRNA-seq, and 971 genes (4%) were unique to snRNA-seq (Supplementary Fig. 5). Considering genes expressed in at least 10% of the cells/nuclei, 4332 genes were detected overall, with 1251 genes (29%) in both datasets. Most (3014 genes, 70%) were exclusive to scRNA-seq, whereas only 67 genes (1.5%) were unique to snRNA-seq (Supplementary Fig. 5). The number of genes detected per annotated cell type (UMI ≥ 3) in each dataset (snRNA-seq or scRNA-seq) and the fraction of barcodes each gene was detected in are provided in Supplementary Table 5. The number of detected genes varied by cell type, ranging from 10,722 to 17,137 genes per cell type in scRNA-seq and 5275 to 16,235 for snRNA-seq (Supplementary Table 6). For example, while scRNA-seq detected 17,096 genes in beta cells, snRNA-seq detected 16,235.

### Robust and novel marker genes in the annotated scRNA-seq and snRNA-seq datasets

Next, to identify robust and novel cell-type-specific markers, we performed a second FindAllMarkers analysis using stricter criteria (log₂FC > 1, expressed in ≥ 50% of barcodes), comparing the ten annotated cell types from the manual annotation (Fig. 2b). As a sensitivity analysis, we excluded clusters 1, 14, and 18 (Fig. 2a), which were likely stressed beta cells, in the beta cell annotation (Supplementary Fig. 6). However, since removing these cell clusters had minimal influence on the marker gene list for beta cells (85% overlap in the marker gene lists; data not shown), we kept them as being annotated as beta cells. The top 10 marker genes for each cell type for scRNA-seq and snRNA-seq are shown in Table 4, while the complete lists are shown in Supplementary Table 7. When comparing the identified marker genes presented in Supplementary Table 7 for scRNA-seq and snRNA-seq for the endocrine cells, the overlap was 8% for beta cells (among the top ten based on adjusted p-values, the following genes were overlapping; *INS, ZNF385D, HADH, MEG3*), 17% for alpha cells (among top ten; *MMP16, FASTL5, GP6,* and *PPP2R2B*), 21% for delta cells (among top ten; *LRFN5, KCTN2, DPYSL3, ERBB4, SST, ADGRL2,* and *THSD7A*), and 14% for gamma cells (among top ten; *CACNA2D3, CHRM,* and *KCNT2*). Canonical endocrine markers *(INS, GCG, SST, PPY*) were expressed in nearly all annotated beta, alpha, delta, and gamma cells (99%, 99%, 99%, and 90%, respectively, expressed defined as UMI ≥ 3)) but at lower levels in nuclei (59%, 58%, 86%, and 46%, respectively (FDR 0.14, Supplementary Fig. 7). However, the canonical endocrine marker genes also had a higher expression in other cell types than their primary cell type for scRNA-seq compared to snRNA-seq, and these canonical marker genes were higher up in the marker gene lists for the “correct” nuclei than for cells (Supplementary Table 6). Interestingly, *ZNF385D* was the top beta cell marker in snRNA-seq and second-ranked in scRNA-seq, with higher expression in beta nuclei than in beta cells (Table 4). *INS* was detected in 99% of beta cells (based on a gene being expressed in a barcode if it had a UMI ≥ 3; the pct.1 column in Table 4 is based on being expressed in a barcode if it had a UMI ≥ 1 as is default in the FindAllMarkers function) but only 59% of beta nuclei (FDR 0.14, Supplementary Fig. 7), whereas *ZNF385D* was detected in 39% of beta cells and 82% of beta nuclei (FDR 0.14, Supplementary Fig. 7). Of note, nuclear transcripts may reflect a snapshot of active transcription, while transcripts in the cytosol may be accumulated over a longer time.

**Table 4:** Top 10 marker genes (ranked according to adjusted p-value) identified by the Seurat FindAllMarkers function for scRNA-seq and snRNA-seq separately. The table lists marker genes per cell type, along with associated statistics (p-value, average log₂ fold change, the percentage of cells/nuclei where the gene is detected (UMI ≥ 1) in the cell type of interest, the percentage of cells/nuclei where the gene is detected in the second group (average across all other cell types than the target cell type). FDR-values are not presented in the table since all are very low – the highest p-value in the list is 6.54 × 10^−61^.

Gene	ScRNA-seq				Gene	SnRNA-seq			
	Full gene name	Average	pct.1^a^	pct.2^a^		Full gene name	Average	pct.1	pct.2
		log2FC^a^					log2FC		
Acinar									
* CTRB2*	Chymotrypsinogen B2	8.5	0.97	0.05	*MECOM*	MDS1 And EVI1 Complex Locus	6.8	0.79	0.02
* PRSS1*	Protease. Serine 1 (Trypsin 1)	7.9	0.92	0.04	*CD44*	CD44 Molecule (Indian Blood Group)	4.4	0.96	0.23
* CTRB1*	Chymotrypsinogen B1	8.2	0.90	0.03	*AC007368.1*	Uncharacterized LOC101927285	7.5	0.75	0.01
* CELA3A*	Chymotrypsin-Like Elastase Family Member 3A	7.7	0.85	0.04	*NIBAN1*	Niban Apoptosis Regulator 1	5.3	0.78	0.06
* PLA2G1B*	Phospholipase A2 Group IB	7.9	0.83	0.04	*ZFP36L1*	ZFP36 Ring Finger Protein Like 1	5.0	0.77	0.08
* CTRC*	Chymotrypsin C (Caldecrin)	7.7	0.78	0.02	*CHRM3*	Cholinergic Receptor Muscarinic 3	3.2	0.78	0.14
* CPA2*	Carboxypeptidase A2	8.2	0.74	0.01	*ZNF704*	Zinc Finger Protein 704	4.2	0.71	0.07
* CPA1*	Carboxypeptidase A1	8.2	0.74	0.01	*SLC25A37*	Solute Carrier Family 25 Member 37	3.7	0.73	0.11
* CPB1*	Carboxypeptidase B1	7.6	0.75	0.02	*TC2N*	Tandem C2 Domains. Nuclear	3.9	0.73	0.12
* SYCN*	Syncollin	8.1	0.66	0.01	*GULP1*	GULP. Engulfment Adaptor PTB Domain Containing 1	2.2	0.82	0.21
Alpha									
* GC*	Group-Specific Component	2.4	0.88	0.14	*GCG*	Glucagon	3.9	0.72	0.03
* IRX2*	Iroquois Homeobox 2	3.7	0.77	0.06	*DSCAM*	Down Syndrome Cell Adhesion Molecule	3.1	0.81	0.18
* F10*	Coagulation Factor X	2.5	0.79	0.08	*MMP16*	Matrix Metallopeptidase 16	4.2	0.71	0.08
* TMEM176B*	Transmembrane Protein 176B	2.7	0.86	0.19	*TTR*	Transthyretin	3.5	0.69	0.08
* FSTL5*	Follistatin-Like 5	3.1	0.76	0.09	*GPC6*	Glypican 6	2.4	0.81	0.21
* TMEM176A*	Transmembrane Protein 176A	2.7	0.82	0.15	*FSTL5*	Follistatin Like 5	3.9	0.66	0.06
* GPC6*	Glypican 6	2.9	0.86	0.19	*SAMD5*	Sterile Alpha Motif Domain Containing 5	2.1	0.84	0.28
* C5orf38*	Chromosome 5 Open Reading Frame 38	3.6	0.70	0.05	*PPP2R2B*	Protein Phosphatase 2 Regulatory Subunit Bbeta	4.4	0.64	0.07
* MMP16*	Matrix Metallopeptidase 16	3.6	0.73	0.09	*SLC35F4*	Solute Carrier Family 35 Member F4	2.7	0.73	0.17
* PPP2R2B*	Protein Phosphatase 2 Regulatory Subunit Bbeta	4.3	0.71	0.07	*AL033504.1*	Uncharacterized LOC101927506	2.2	0.79	0.25
Alpha + beta									
* LINC01482*	Long Intergenic Non-Protein Coding RNA 1482	5.9	0.70	0.05	*PDE4C*	Phosphodiesterase 4C	4.9	0.80	0.08
* AL163541.1*	Uncharacterized LOC101927759	6.1	0.67	0.03	*LINC02245*	Long Intergenic Non-Protein Coding RNA 2245	4.2	0.75	0.15
* DNAH2*	Dynein Axonemal Heavy Chain 2	6.5	0.65	0.02	*DNAH12*	Dynein Axonemal Heavy Chain 12	4.3	0.74	0.15
* EGLN3*	Egl-9 Family Hypoxia Inducible Factor 3	5.3	0.75	0.13	*NUP210L*	Nucleoporin 210 Like	4.6	0.63	0.06
* NUP210L*	Nucleoporin 210 Like	4.4	0.73	0.12	*AP000446.1*	Uncharacterized LOC101927759	4.1	0.67	0.12
* AP000446.1*	Uncharacterized LOC101928267	5.3	0.71	0.10	*SGO1-AS1*	SGO1 Antisense RNA 1	4.5	0.68	0.13
* TULP2*	Tubby Like Protein 2	4.8	0.62	0.04	*TPH2*	Tryptophan Hydroxylase 2	5.9	0.57	0.03
* SNX31*	Sorting Nexin 31	5.4	0.58	0.03	*LINC01482*	Long Intergenic Non-Protein Coding RNA 1482	5.3	0.58	0.04
* PDE4C*	Phosphodiesterase 4C	5.7	0.59	0.04	*AL138828.1*	Uncharacterized LOC101927867	4.0	0.64	0.10
* AC092422.1*	Uncharacterized LOC101928546	6.6	0.56	0.03	*NLRC5*	NLR Family CARD Domain Containing 5	4.8	0.67	0.14
Beta									
* INS*	Insulin	4.3	0.99	0.51	*ZNF385D*	Zinc Finger Protein 385D	5.8	0.91	0.05
* ZNF385D*	Zinc Finger Protein 385D	4.3	0.55	0.12	*TRPM3*	Transient Receptor Potential Cation Channel Subfamily M Member 3	3.1	0.82	0.13
* IAPP*	Islet Amyloid Polypeptide	4.3	0.80	0.37	*NRG1*	Neuregulin 1	2.7	0.81	0.13
* HADH*	Hydroxyacyl-CoA Dehydrogenase	2.9	0.63	0.26	*CASR*	Calcium-Sensing Receptor	3.1	0.82	0.15
* MEG3*	Maternally Expressed 3 (non-protein coding)	2.4	0.58	0.25	*MEG3*	Maternally Expressed 3 (non-protein coding)	1.3	0.88	0.24
* UCHL1*	Ubiquitin C-Terminal Hydrolase L1	1.9	0.70	0.45	*HDAC9*	Histone Deacetylase 9	2.6	0.91	0.31
* PKIB*	Protein Kinase (cAMP-Dependent. Catalytic) Inhibitor Beta	1.8	0.55	0.23	*PPM1E*	Protein Phosphatase. Mg2+/Mn2+Dependent 1E	3.5	0.71	0.11
* FXYD2*	FXYD Domain Containing Ion Transport Regulator 2	2.5	0.62	0.41	*DOCK10*	Dedicator of Cytokinesis 10	4.0	0.66	0.06
* HDAC9*	Histone Deacetylase 9	1.5	0.51	0.25	*MEG8*	Maternally Expressed 8 (non-protein coding)	1.8	0.78	0.20
* ERO1B*	ERO1-Like Beta Oxidase	1.3	0.66	0.63	***INS***	**Insulin**	3.1	0.61	0.06
Delta									
* LRFN5*	Leucine Rich Repeat And Fibronectin Type III Domain Containing 5	5.9	0.81	0.06	***SST***	**Somatostatin**	7.7	0.89	0.00
* KCNT2*	Potassium Sodium-Activated Channel Subfamily T Member 2	4.9	0.57	0.04	*LRFN5*	Leucine Rich Repeat And Fibronectin Type III Domain Containing 5	6.2	0.90	0.04
* DPYSL3*	Dihydropyrimidinase Like 3	3.6	0.59	0.08	*KCNT2*	Potassium Sodium-Activated Channel Subfamily T Member 2	5.0	0.82	0.06
* ERBB4*	Erb-B2 Receptor Tyrosine Kinase 4	4.8	0.57	0.08	*ADGRL2*	Adhesion G Protein-Coupled Receptor L2	3.5	0.63	0.09
* SST*	Somatostatin	6.4	1.00	0.73	*DPYSL3*	Dihydropyrimidinase Like 3	3.2	0.58	0.09
* ADGRL2*	Adhesion G Protein-Coupled Receptor L2	2.6	0.66	0.14	*ERBB4*	Erb-B2 Receptor Tyrosine Kinase 4	4.6	0.54	0.06
* RBP4*	Retinol Binding Protein 4	4.5	0.88	0.27	*KCTD8*	Potassium Channel Tetramerization Domain Containing 8	2.5	0.78	0.27
* SLC38A1*	Solute Carrier Family 38 Member 1	2.1	0.64	0.13	*TENM3*	Teneurin Transmembrane Protein 3	2.6	0.82	0.35
* KCTD8*	Potassium Channel Tetramerization Domain Containing 8	3.8	0.71	0.18	*RALYL*	RALY RNA Binding Protein Like	2.2	0.75	0.25
* THSD7A*	Thrombospondin Type 1 Domain Containing 7A	3.5	0.64	0.14	*THSD7A*	Thrombospondin Type 1 Domain Containing 7A	2.4	0.60	0.17
Ductal									
* KRT19*	Keratin 19	6.9	0.79	0.04	*ABCC3*	ATP Binding Cassette Subfamily C Member 3	4.8	0.81	0.06
* ANXA3*	Annexin A3	5.7	0.77	0.04	*THSD4*	Thrombospondin Type 1 Domain Containing 4	5.2	0.77	0.05
* KRT7*	Keratin 7	5.3	0.75	0.03	*SVIL*	Supervillin	4.0	0.91	0.19
* LGALS3*	Galectin 3	3.9	0.84	0.14	*SMAD3*	SMAD Family Member 3	3.6	0.90	0.21
* MET*	MET Proto-Oncogene. Receptor Tyrosine Kinase	4.7	0.74	0.06	*MYO1E*	Myosin IE	3.5	0.78	0.13
* LCN2*	Lipocalin 2	6.2	0.72	0.03	*ARHGAP26*	Rho GTPase Activating Protein 26	3.7	0.83	0.20
* MYOF*	Myoferlin	4.6	0.78	0.10	*LAMC2*	Laminin Subunit Gamma 2	5.2	0.69	0.06
* S100A14*	S100 Calcium Binding Protein A14	7.5	0.68	0.01	*YAP1*	Yes Associated Protein 1	3.5	0.70	0.09
* TINAGL1*	Tubulointerstitial Nephritis Antigen Like 1	5.3	0.73	0.06	*MYOF*	Myoferlin	5.4	0.63	0.03
* EPS8*	Epidermal Growth Factor Receptor Pathway Substrate 8	3.7	0.81	0.16	*PMEPA1*	Prostate Transmembrane Protein. Androgen Induced 1	6.4	0.62	0.02
Endothelial									
* PCAT19*	Prostate Cancer Associated Transcript 19 (non-protein coding)	6.4	0.90	0.01	*ADGRL4*	Adhesion G Protein-Coupled Receptor L4	9.9	0.74	0.00
* FLT1*	Fms Related Receptor Tyrosine Kinase 1	7.8	0.89	0.02	*PTPRB*	Protein Tyrosine Phosphatase Receptor Type B	9.1	0.69	0.00
* ADGRL4*	Adhesion G Protein-Coupled Receptor L4	8.0	0.87	0.01	*FLT1*	Fms Related Receptor Tyrosine Kinase 1	8.5	0.63	0.01
* IFI27*	Interferon Alpha Inducible Protein 27	5.4	0.88	0.04	*ERG*	ETS Transcription Factor ERG	8.3	0.57	0.01
* PODXL*	Podocalyxin Like	7.2	0.87	0.03	*PLVAP*	Plasmalemma Vesicle Associated Protein	8.9	0.55	0.00
* LAMA4*	Laminin Subunit Alpha 4	6.3	0.84	0.03	*CD93*	CD93 Molecule	10.7	0.53	0.00
* CALCRL*	Calcitonin Receptor Like Receptor	7.0	0.83	0.02	*CALCRL*	Calcitonin Receptor Like Receptor	8.0	0.61	0.01
* PLVAP*	Plasmalemma Vesicle Associated Protein	7.8	0.83	0.02	*LAMA4*	Laminin Subunit Alpha 4	6.7	0.57	0.01
* GNG111*	G Protein Subunit Gamma 11	4.3	0.89	0.08	*PXDN*	Peroxidasin	6.4	0.55	0.01
* PECAM1*	Platelet And Endothelial Cell Adhesion Molecule 1	6.3	0.83	0.04	*EMP1*	Epithelial Membrane Protein 1	6.4	0.61	0.02
Gamma									
* NPFFR2*	Neuropeptide FF Receptor 2	6.1	0.51	0.01	*CACNA2D3*	Calcium Voltage-Gated Channel Auxiliary Subunit Alpha2delta 3	4.6	0.77	0.06
* CACNA2D3*	Calcium Voltage-Gated Channel Auxiliary Subunit Alpha2delta 3	5.0	0.80	0.05	*CHRM3*	Cholinergic Receptor Muscarinic 3	2.8	0.86	0.19
* EYA4*	EYA Transcriptional Coactivator And Phosphatase 4	5.4	0.54	0.03	*THSD7A*	Thrombospondin Type-1 Domain-Containing Protein 7A	2.8	0.82	0.17
* GPC5-AS1*	GPC5 Antisense RNA 1 (non-protein coding)	4.4	0.52	0.03	*PPY*	Pancreatic Polypeptide	7.1	0.64	0.00
* CHRM3*	Cholinergic Receptor Muscarinic 3	4.9	0.90	0.14	*GPC6*	Glypican 6	2.8	0.93	0.30
* DPYSL31*	Dihydropyrimidinase Like 3	4.1	0.74	0.09	*SNTG1*	Syntrophin Gamma 1	3.0	0.86	0.26
* SERTM1*	Serine Rich And Transmembrane Domain Containing 1	3.8	0.51	0.04	*TENM2*	Teneurin Transmembrane Protein 2	4.2	0.66	0.08
* KCNC2*	Potassium Voltage-Gated Channel Subfamily C Member 2	3.7	0.63	0.07	*KCTD16*	Potassium Channel Tetramerization Domain Containing 16	3.7	0.69	0.12
* KCNT21*	Potassium Sodium-Activated Channel Subfamily T Member 2	3.7	0.55	0.05	*RALYL*	RALY RNA Binding Protein Like	2.7	0.80	0.25
* AC092691.1*	Uncharacterized LOC101927506 (non-protein coding)	4.0	0.58	0.06	*KCNT2*	Potassium Sodium-Activated Channel Subfamily T Member 2	2.9	0.61	0.07
Immune									
* JUN*	Jun Proto-Oncogene. AP-1 Transcription Factor Subunit	5.7	0.89	0.07	*ITGAX*	Integrin Subunit Alpha X	10.2	0.76	0.00
* SRGN*	Serglycin	8.7	0.79	0.01	*ZEB21*	Zinc Finger E-Box Binding Homeobox 2	7.7	0.74	0.01
* LAPTM5*	Lysosomal Protein Transmembrane 5	7.7	0.67	0.01	*CSF2RA*	Colony Stimulating Factor 2 Receptor Alpha Subunit	8.9	0.62	0.01
* LCP1*	Lymphocyte Cytosolic Protein 1	6.8	0.67	0.03	*DOCK2*	Dedicator of Cytokinesis 2	8.5	0.61	0.01
* SAMSN1*	SAM Domain. SH3 Domain And Nuclear Localization Signals 1	8.6	0.65	0.01	*KYNU*	Kynureninase	5.8	0.62	0.02
* DOCK2*	Dedicator of Cytokinesis 2	8.3	0.63	0.01	*FAM49A*	Family With Sequence Similarity 49 Member A	7.6	0.60	0.01
* PRKCB*	Protein Kinase C Beta	7.2	0.63	0.01	*PIK3R5*	Phosphoinositide-3-Kinase Regulatory Subunit 5	10.4	0.58	0.00
* ARHGAP15*	Rho GTPase Activating Protein 15	6.4	0.65	0.04	*PTPRC*	Protein Tyrosine Phosphatase Receptor Type C	11.5	0.54	0.00
* DOCK8*	Dedicator of Cytokinesis 8	9.6	0.62	0.01	*CD109*	CD109 Molecule	7.6	0.53	0.01
* PTPRC*	Protein Tyrosine Phosphatase Receptor Type C	9.4	0.62	0.01	*ARHGAP15*	Rho GTPase Activating Protein 15	8.4	0.52	0.01
Stellate									
* COL6A2*	Collagen Type VI Alpha 2 Chain	6.5	0.89	0.05	*COL4A2*	Collagen Type IV Alpha 2 Chain	7.4	0.77	0.02
* BGN*	Biglycan	6.2	0.80	0.01	*COL6A2*	Collagen Type VI Alpha 2 Chain	9.4	0.76	0.01
* LGALS1*	Galectin 1	4.0	0.86	0.09	*COL4A1*	Collagen Type IV Alpha 1 Chain	7.1	0.74	0.03
* C11orf96*	Chromosome 11 Open Reading Frame 96	5.7	0.74	0.02	*CDH11*	Cadherin 11	10.0	0.66	0.00
* MFGE8*	Milk Fat Globule-EGF Factor 8 Protein	4.8	0.76	0.05	*ZEB2*	Zinc Finger E-Box Binding Homeobox 2	6.2	0.63	0.01
* BASP1*	Brain Abundant Membrane Attached Signal Protein 1	4.4	0.75	0.05	*ADAMTS12*	ADAM Metallopeptidase With Thrombospondin Type 1 Motif 12	7.2	0.63	0.02
* RND3*	Rho Family GTPase 3	4.7	0.83	0.13	*GLI2*	GLI Family Zinc Finger 2	9.8	0.58	0.00
* COL6A1*	Collagen Type VI Alpha 1 Chain	5.8	0.79	0.10	*EBF1*	Early B Cell Factor 1	5.7	0.59	0.02
* SPARC*	Secreted Protein Acidic And Cysteine Rich	3.4	0.75	0.06	*ZFPM2*	Zinc Finger Protein. FOG Family Member 2	7.1	0.57	0.01
* YBX3*	Y-Box Binding Protein 3	2.9	0.81	0.15	*EDNRA*	Endothelin Receptor Type A	9.1	0.55	0.00

Significant values are in bold.

^a^log2FC: log fold-change of the average expression between the two groups.

Pct.1: The percentage of cells where the feature is detected in the cell type of interest.

Pct.2: The percentage of cells where the feature is detected in the second group (average across all other genes, not for a specific cell type).

For exocrine cells, the overlap in marker genes for snRNA-seq and scRNA-seq was only 4% for acinar cells (none in the top 10) and 26% for ductal cells (among the top ten; *MYOF*). The canonical marker for acinar cells, *PRSS1,* was the second-ranked marker for acinar cells in scRNA-seq but absent in the snRNA-seq marker gene list and was detected in 76% of acinar cells but only in 18% of nuclei (FDR 0.14, Supplementary Fig. 7). Another canonical acinar marker, *REG1A*, was in the marker gene list for both cells and nuclei but was detected at different fractions; 94% of the cells and 39% of the nuclei (FDR 0.25, Supplementary Fig. 7). *MECOM* and *CD44* were the top marker genes for acinar cells.

Similarly, the canonical marker for ductal cells, *KRT19*, was the top marker for ductal cells in scRNA-seq but not present in the snRNA-seq marker gene list. *KRT19* was detected in 63% of the annotated duct cells and 2.5% of the annotated duct cell nuclei (FDR 0.14, Supplementary Fig. 7). *ABCC3* and *THSD4* were the top marker genes for ductal cells.

The overlap in marker genes was 15% for endothelial cells (among the top ten; *FLT1, ADGRL4, LAMA4, CALCRL, PLVAP*), 24% for immune cells (among the top ten; *DOCK2, ARHGAP15, PTPRC*), and 21% for stellate cells (among the top ten; *COL6A2*).

To further explore the robustness of marker genes for snRNA-seq, we compared our snRNA-seq marker gene lists (Supplementary Table 7) with the marker genes identified in snRNA-seq data from human islets by Kang et al.^25^. Despite some differences in selection criteria—our study applied cutoffs on log2FC > 1 and expression in ≥ 50% of barcodes, UMI > 1 per gene (Seurat FindAllMarkers), while Kang et al. used log₂FC > 1.5, p ≈ 0, and no minimum expression frequency—a substantial proportion, of their marker genes were also present in our dataset. For beta cells, *ZNF385D* and *TRPM3*, two of the four top marker genes from Kang et al., were also present among our marker genes, whereas their remaining top markers, *LRFN2* and *PLUT,* were expressed in less than 50% of beta nuclei and were therefore excluded from our analysis. However, *LRFN2* was identified in our initial cluster-based marker analysis, applying a less strict cutoff, and was associated with most clusters later annotated as beta cells (Supplementary Table 1). In total, 103 (94%) beta cell marker genes overlapped between our study and the study by Kang et al. (Supplementary Table 8).

For alpha cells, none of the four top marker genes from Kang et al. (*PTPRT, FAP*, *PDK4, LOXL4*) were identified in our snRNA-seq marker gene list, likely due to an expression frequency < 50% in nuclei and our stringent cut off criteria. However, *PTPRT, FAP*, and *PDK4* were identified as snRNA-seq marker genes in our initial cluster-based marker analysis requiring expression in a lower fraction of nuclei and were associated with most clusters later annotated as alpha cells. In total, 60 (61%) alpha cell marker genes overlapped between our study and the study by Kang et al. (Supplementary Table 8).

For delta cells, marker genes *LRFN5, KCNT2,* and *ERBB4* from Kang et al.^25^ were among the top 10 markers in our dataset. The fourth marker gene from Kang et al., *ADARB2,* had an expression frequency below 50%, but it was detected as a delta marker in our initial analysis with a lower threshold. In total, 28 (54%) delta cell marker genes overlapped between our study and the study by Kang et al. (Supplementary Table 8).

For gamma cells, *CACNA2D3, THSD7A*, and *CNTNAP5*, three top markers from Kang et al. were identified in our marker gene list. In total, 16 (20%) gamma cell marker genes overlapped between our study and the study by Kang et al. (Supplementary Table 8).

**Fig. 3:**
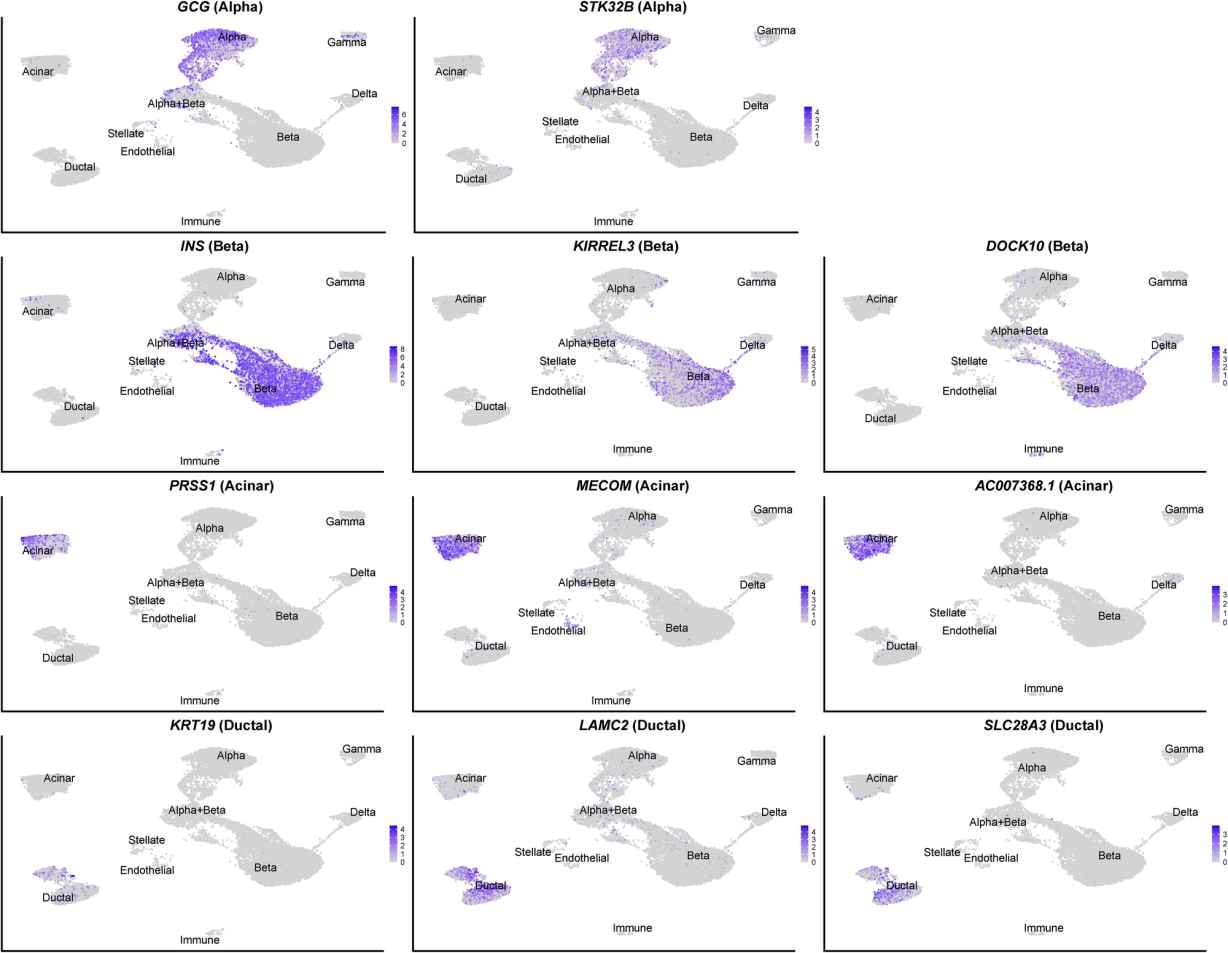
Uniform Manifold Approximation and Projection (UMAP) feature plots showing the expression of selected canonical marker genes and novel snRNA-seq marker genes for the snRNA-seq data. Each panel displays a single gene, along with its corresponding target cell type within brackets. Expression intensity is indicated by color with a gradient shown on the right side of the panel. Cell types are labeled in the UMAP for reference.

Overall, the relatively high overlap between cell-type-specific genes in our data set and those of Kang et al. supports using specific marker genes for snRNA-seq data rather than marker genes from scRNA-seq analyses. Additionally, we propose a set of additional, novel snRNA-seq marker genes, to further improve cell type prediction in snRNA-seq. Some novel snRNA-seq marker genes are shown in Fig. 3 together with the canonical marker genes for that cell type: *DOCK10* and *KIRREL3* for beta cells, *STK32B* for alpha cells, *MECOM* and *AC007368.1* for acinar cells, and *LAMC2* and *SLC28A3* for ductal cells. We did not find specific snRNA-seq marker genes for delta or gamma cells. Expression levels of selected canonical marker genes and snRNA-seq markers per cell type at the donor level are visualized in Supplementary Fig. 8. In addition, to compare the expression of canonical marker genes and snRNA-seq markers at different annotation methods for snRNA-seq, dotplots are shown in Supplementary Fig. 9.

In addition, Lee et al.^41^ recently identified two clusters of nuclei potentially transitioning from alpha to beta, which they called c11 and c13. We further investigated whether our alpha + beta cell cluster had similarities with these c11 and c13 clusters. In Lee et al.^41^, it was found that at the cluster level, c11 cells exhibited high expression of both *GCG* and *INS*, but when looking at per-cell expression, most cells expressed high levels of *GCG*, while a subset exhibited elevated *INS*, but none was double-positive for *INS* and *GCG.* We found a similar pattern in our snRNA-seq data, where nuclei in the alpha + beta cluster expressed high levels of *GCG* or *INS*, but very few nuclei expressed both *GCG* and *INS* at high levels (Supplementary Fig. 9). A tendency to a similar pattern was seen for scRNA-seq, although many cells expressed both *GCG* and *INS* at high levels. Lee et al.^41^ also identified Tryptophan hydroxylase 2 (*TPH2*) as a marker gene for c11 cells in all donors analyzed, and we also found *TPH2* to be a marker gene in both scRNA-seq and snRNA-seq (Table 3, Supplementary Table 7).

To discover genes that are differently expressed between scRNA-seq and snRNA-seq in a cell type, we performed a FindAllMarkers analysis. A list of genes, with higher expression in scRNA-seq compared to snRNA-seq, is shown in Supplementary Table 9, while a list of genes, with higher expression in snRNA-seq compared to scRNA-seq, is shown in Supplementary Table 10. The novel snRNA-seq marker genes *DOCK10* and *KIRREL3* for beta cells, *STK32B* for alpha cells, *MECOM* and *AC007368.1* for acinar cells, and *LAMC2* and *SLC28A3* for ductal cells, were all also in the list of DEGs positively expressed in snRNA-seq compared to scRNA-seq, indicating that these marker genes may be specific for snRNA-seq, possibly due to detection bias. Expression levels of these marker genes per cell type at the donor level and per modality are visualized in Supplementary Fig. 8. For genes that were higher expressed in scRNA-seq compared to snRNA-seq there were, as expected, a large amount of ribosomal (RPL) and mitochondrial (MT−) genes, but also well-known canonical marker genes such as *INS* (beta), *GCG* (alpha), *SST* (delta), *PPY* (gamma), *PSSR1* (acinar), *REG1A* (acinar) and *KRT19* (ductal).

### *ZNF385D* is needed for normal insulin secretion

**Fig. 4:**
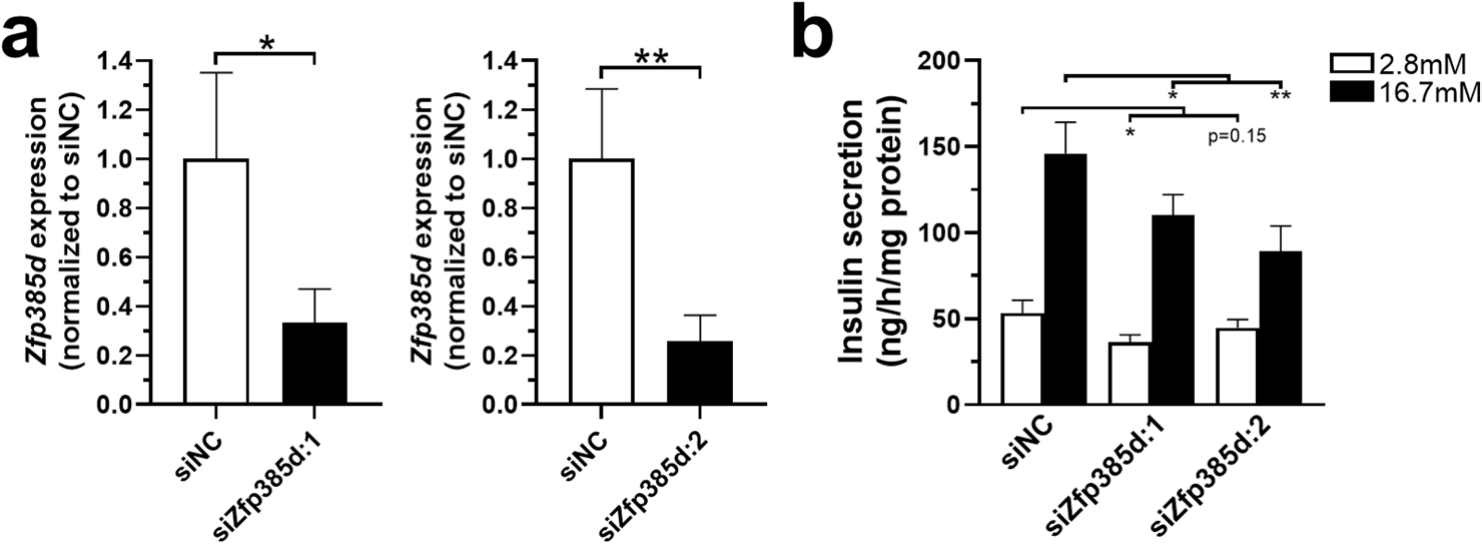
Loss of *Zfp385d* results in perturbed insulin secretion in 832/13 INS1 cells. The panels show (**a**) qPCR quantification of siRNA-mediated knockdown of *Zfp385d* (n = 5) (siZfp385d:1) and 6 (siZfp385d:2)). (**b**) Beta cells deficient for *Zfp385d* exhibit perturbed glucose-stimulated insulin secretion (n = 6). siNC: negative control siRNA. *p < 0.05 and **p < 0.01, based on one-tailed (**a**) and two-tailed (**b**) paired t-tests.

We identified *ZNF385D* as the top- and second-ranked beta cell marker in snRNA-seq and scRNA-seq, respectively. Genes that are selectively expressed in a specific cell type are strong candidates for having important functional roles in said cell type. For beta cells, the main function is glucose-stimulated insulin secretion. Hence, to examine whether *ZNF385D* has a role in insulin secretion, we used two different siRNAs to knock down its rat homologue *Zfp385d* in 832/13 INS1 cells. This resulted in ~ 70% knockdown of *Zfp385d* with either siRNA (Fig. 4a). Importantly, loss of *Zfp385d* perturbed insulin secretion at both basal and high glucose (Fig. 4b).

## Discussion

This study systematically compared scRNA-seq and snRNA-seq datasets from fresh and frozen human pancreatic islets obtained from the same organ donors. Our findings provide insight into differences in predicted cell type composition, reference-based annotation accuracy, gene detection, and marker gene identification between these two RNA sequencing protocols. Importantly, our analysis shows that scRNA-seq references are insufficient for cell type annotation of snRNA-seq data. We identified novel snRNA-seq markers, which can improve snRNA-seq-based annotation and can be used for future studies.

While the expected pancreatic cell populations were identified with both methods, epsilon cells were undetected (as previously reported in 10x-based studies)^19^ for all annotation methods; however, it was unclear whether there were any epsilon cells according to the HPAP annotation, since the HPAP annotation had a combined gamma and epsilon cell type. Although scRNA-seq and snRNA-seq samples originated from the same donors, we found rather large differences in predicted cell type proportions between the two methods, particularly for acinar, stellate, immune, and endothelial cell types, but also for beta cells. These discrepancies may reflect differences in RNA recovery due to transcriptional changes during dissociation and freezing^20^. It has previously been reported that snRNA-seq recovers more attached cell types, whereas scRNA-seq is biased towards immune cell types^20,21,33,42^. Additionally, previous studies demonstrated that islets obtained from the same donor are heterogenous and show differences in cell composition^43,44^. Subsequently, although it is a strength that we used a paired analysis, where islets from the same four donors were used for scRNA-seq and snRNA-seq, we cannot exclude that differences in cell composition in the islets used for the two methods affect our results. Hence, although the large number of islets analysed should minimize this risk, it is possible that the differences seen between the two methods may partly be due to differences in cell composition in islets obtained from the same donor.

scRNA-seq and snRNA-seq differed in the number of detected genes, probably due to lower nuclear mRNA content and the absence of cytoplasmic transcripts^21,24,25^. Across cell types, gene detection was notably lower in immune, endothelial, and stellate cells in snRNA-seq, leading to a lower overlap between genes detected in nuclei and whole cells compared to other cell types. Additional factors that may contribute to differences in detected transcripts between the two methods include transcriptional degradation and nuclear transcript localization^19,31^. Furthermore, Xie et al. compared five different nuclei-isolation methods prior to snRNA-seq, and showed that there were differential enrichment of transcripts belonging to different functional classes in snRNA-seq compared to scRNA-seq^26^. It should also be noted that, e.g., *INS*, *GCG*, *SST*, and *PPY*, were not the top detected genes in the snRNA-seq data. Previously, Kang et al.^25^ discussed the fact that the most abundant mRNAs in human endocrine cells do not reflect the most profused pre-RNAs in those cells, potentially because a large proportion of steady-state cytoplasmic mRNA in beta cells is relatively stable, e.g., *INS* mRNA awaiting translation in response to glucose^25,45^. These technical and biological differences highlight the importance of identifying snRNA-seq-specific markers, as we report here, rather than relying solely on canonical scRNA-seq markers. To evaluate the performance of existing scRNA-seq-based annotation tools on snRNA-seq data, we applied reference-based annotation using Azimuth and Seurat’s label transfer with CCA integration. Several annotation methods are available for scRNA-seq. However, the Seurat label transfer, which was used for HPAP and on which Azimuth is also based, is often more robust across modalities^36,46^. Thus, we hypothesized this is one of the more accurate methods for annotating snRNA-seq data. The annotation quality is also dependent on the quality of the reference dataset. The HPAP dataset is relatively new and deeply sequenced^30^, while the datasets in Azimuth’s *pancreasref*^13–15,27–29^ are older and less deeply sequenced, which could influence the results.

The three annotation methods showed substantial variation for the snRNA-seq compared to the scRNA-seq data, based on predicted cell type proportions, pair-wise overlap of cell types, and prediction and mapping scores. This was especially valid for the Azimuth annotation and its annotation of acinar, delta, and gamma cells. The predicted fractions of delta and gamma cells were around 2 (delta) and 4 (gamma) times higher for Azimuth compared to the manual annotation and 4 (delta) and 9 (gamma) times higher for Azimuth compared to HPAP, while the predicted fractions of acinar cells were around 40 times higher for manual annotation and HPAP compared to Azimuth. The HPAP annotation was generally more concordant with the manual annotation than Azimuth was. However, a limitation is that the different annotations differ in their cell type definitions—for example, HPAP includes an alpha + beta category, while Azimuth does not—which can bias the classification of transitional clusters and partly explain the lower concordance observed for snRNA-seq for Azimuth. Some pair-wise comparisons (gamma and delta) showed a low agreement in annotated cell type, also when comparing the manual annotation and the HPAP annotation, and the prediction scores for these cell types were low. Together, this highlights that reference-based annotations using scRNA-seq references are suitable for scRNA-seq data but not for snRNA-seq data, suggesting that scRNA-seq-derived reference datasets may not fully capture the transcriptional landscape of nuclei. These findings underscore the need for snRNA-seq-specific reference datasets and improved annotation strategies to enhance cell type classification accuracy in snRNA-seq.

Given the challenges in applying scRNA-seq reference-based annotations to snRNA-seq data, we sought to identify snRNA-seq-specific marker genes for improving cell type annotation in human pancreatic islets. By applying stringent criteria, we identified novel specific marker genes. The overlap between marker genes for scRNA-seq and snRNA-seq was relatively low, which further merits the identification of novel snRNA-seq-specific marker genes for improving cell type annotation in human pancreatic islets. Our findings confirm that many of the marker genes from Kang et al.^25^ are also marker genes for our snRNA-seq analysis, though some were below our expression frequency threshold (50%). Overall, the relatively high overlap between cell-type-specific genes in our data set, which included four donors, and the study by Kang et al., which included three donors, supports using specific marker genes for snRNA-seq data. Importantly, we also found a set of novel marker genes for different cell types using snRNA-seq islet data, such as, e.g., *DOCK10* and *KIRREL3* (beta), *STK32B* (alpha), *MECOM* and *AC007368.1* (acinar), and *LAMC2* and *SLC28A3* (ductal). These genes exhibited higher expression in snRNA-seq compared to scRNA-seq, indicating that these marker genes may be selective for snRNA-seq, possibly due to detection bias. Additionally, Kang et al. found the scRNA-seq canonical markers for acinar and ductal cells to be specific marker genes also for snRNA-seq, which we could not confirm in the present study since the canonical marker genes were lowly expressed in our snRNA-seq dataset. Other novel marker genes, such as those mentioned above, were stronger snRNA-seq markers. Lee et al.^41^ recently identified two clusters of nuclei potentially transitioning from alpha to beta. For the alpha + beta annotation in nuclei, we saw some similar results as in Lee et al.^41^, and further studies are merited to investigate this type of cells and its potential transition from alpha to beta.

We confirm that *ZNF385D* is a highly specific marker gene for beta cells and its expression is needed for normal insulin secretion.

This study has some strengths and limitations. It is mainly an exploratory study with a limited number of donors (n = 4) and no experimental validation data set, although we compared our results to data from three islet donors in a previous study, supporting the robustness of our results^25^. However, future validations of the marker genes found in our snRNA-seq data-sets should be performed using the same as well as orthogonal methods. Strengths of the study are that we compared scRNA-seq and snRNA-seq data from fresh and frozen islets from the same human donors. Additionally, we thoroughly compared three annotation methods for islet cell types, supporting the need for different methods for the analysis of sc- and snRNA-seq data.

## Conclusions

Our findings highlight differences in scRNA-seq and snRNA-seq protocols regarding identification of cell type composition, reference-based annotation accuracy, gene detection, and marker gene identification. We conclude that existing scRNA-seq-based references and annotation tools are suboptimal for snRNA-seq, and annotation of snRNA-seq datasets should thus be based on snRNA-seq reference datasets or snRNA-seq-specific marker genes, including the novel marker genes discovered in our study.

## Supplementary Information

Supplementary Information 1.

Supplementary Information 2.

Supplementary Information 3.

Supplementary Information 4.

Supplementary Information 5.

Supplementary Information 6.

Supplementary Information 7.

Supplementary Information 8.

Supplementary Information 9.

## Data Availability

The datasets (genome-wide raw sequencing and individual-level clinical data) generated and/or analysed during the current study are not publicly available due to EU and national legislation, but meta-data and look-ups are available upon reasonable request from the corresponding author. To request access, use the form at https://www.ludc.lu.se/resources/repository. Sc- and sn-RNA sequencing data from human pancreatic islets (accession number LUDC2025.03.1) are deposited in the Lund University Diabetes Centre repository (https://www.ludc.lu.se/resources/repository). No custom code was generated for this study. All analyses were performed using publicly available R packages, including Seurat (v4.4.0) and other standard tools, with only minor modifications to default parameters. The analysis was conducted following established workflows described in the package documentation. Because no original code was created, scripts are not shared. Any additional information required to reanalyze the data reported in this paper is available from the lead contact upon request.

## Abbreviations

CCA: Canonical correlation analysis
DEG: Differentially expressed gene
FC: Fold change
FDR: False discovery rate
*GCG*: Glucagon HPAP Human Pancreas Analysis Program
*INS*: Insulin
PCA: Principal component analysis
scRNA-seq: Single-cell RNA-sequencing
snRNA-seq: Single-nuclei RNA-sequencing
T2D: Type 2 diabetes
UMAP: Uniform manifold approximation and projection
UMI: Unique molecular identifier

## References

[1] Wewer Albrechtsen, N. J. et al. The liver-alpha-cell axis and type 2 diabetes. *Endocr. Rev.* **40**, 1353–1366. 10.1210/er.2018-00251 (2019). 30920583 10.1210/er.2018-00251

[2] Taborsky, G. J. Jr. Evidence of a paracrine role for pancreatic somatostatin in vivo. *Am. J. Physiol.* **245**, E598-603. 10.1152/ajpendo.1983.245.6.E598 (1983). 6140853 10.1152/ajpendo.1983.245.6.E598

[3] Hoffman, E. G., D’Souza, N. C., Liggins, R. T. & Riddell, M. C. Pharmacologic inhibition of somatostatin receptor 2 to restore glucagon counterregulation in diabetes. *Front. Pharmacol.* **14**, 1295639. 10.3389/fphar.2023.1295639 (2023). 38298268 10.3389/fphar.2023.1295639 PMC10829877

[4] Wierup, N., Sundler, F. & Heller, R. S. The islet ghrelin cell. *J. Mol. Endocrinol.* **52**, R35-49. 10.1530/JME-13-0122 (2014). 24049065 10.1530/JME-13-0122

[5] International Diabetes Federation. IDF Diabetes Atlas, 11th edn. Brussels, Belgium: International Diabetes Federation, 2025

[6] Bacos, K. et al. Type 2 diabetes candidate genes, including PAX5, cause impaired insulin secretion in human pancreatic islets. *J. Clin. Invest.*10.1172/JCI163612 (2023). 36656641 10.1172/JCI163612 PMC9927941

[7] Dayeh, T. et al. Genome-wide DNA methylation analysis of human pancreatic islets from type 2 diabetic and non-diabetic donors identifies candidate genes that influence insulin secretion. *PLoS Genet.* **10**, e1004160. 10.1371/journal.pgen.1004160 (2014). 24603685 10.1371/journal.pgen.1004160 PMC3945174

[8] Ronn, T. et al. Genes with epigenetic alterations in human pancreatic islets impact mitochondrial function, insulin secretion, and type 2 diabetes. *Nat. Commun.* **14**, 8040. 10.1038/s41467-023-43719-9 (2023). 38086799 10.1038/s41467-023-43719-9 PMC10716521

[9] Solimena, M. et al. Systems biology of the IMIDIA biobank from organ donors and pancreatectomised patients defines a novel transcriptomic signature of islets from individuals with type 2 diabetes. *Diabetologia* **61**, 641–657. 10.1007/s00125-017-4500-3 (2018). 29185012 10.1007/s00125-017-4500-3 PMC5803296

[10] Volkov, P. et al. Whole-genome Bisulfite sequencing of human pancreatic islets reveals novel differentially methylated regions in type 2 diabetes pathogenesis. *Diabetes* **66**, 1074–1085. 10.2337/db16-0996 (2017). 28052964 10.2337/db16-0996

[11] Wigger, L. et al. Multi-omics profiling of living human pancreatic islet donors reveals heterogeneous beta cell trajectories towards type 2 diabetes. *Nat. Metab.* **3**, 1017–1031. 10.1038/s42255-021-00420-9 (2021). 34183850 10.1038/s42255-021-00420-9

[12] Ewald, J. D. et al. HumanIslets.com: Improving accessibility, integration, and usability of human research islet data. *Cell Metab.* **37**, 7–11. 10.1016/j.cmet.2024.09.001 (2025). 39357523 10.1016/j.cmet.2024.09.001 PMC12577030

[13] Lawlor, N. et al. Single-cell transcriptomes identify human islet cell signatures and reveal cell-type-specific expression changes in type 2 diabetes. *Genome Res.* **27**, 208–222. 10.1101/gr.212720.116 (2017). 27864352 10.1101/gr.212720.116 PMC5287227

[14] Segerstolpe, A. et al. Single-cell transcriptome profiling of human pancreatic islets in health and type 2 diabetes. *Cell Metab.* **24**, 593–607. 10.1016/j.cmet.2016.08.020 (2016). 27667667 10.1016/j.cmet.2016.08.020 PMC5069352

[15] Xin, Y. et al. Single-cell RNA sequencing and analysis of human pancreatic islets. *J. Vis. Exp.*10.3791/59866 (2019). 31380847 10.3791/59866

[16] Fang, Z. et al. Single-cell heterogeneity analysis and CRISPR screen identify key beta-cell-specific disease genes. *Cell Rep.* **26**, 3132-3144.e3137. 10.1016/j.celrep.2019.02.043 (2019). 30865899 10.1016/j.celrep.2019.02.043 PMC6573026

[17] Bosi, E. et al. Integration of single-cell datasets reveals novel transcriptomic signatures of beta-cells in human type 2 diabetes. *NAR Genom. Bioinform.* **2**, Iqaa097. 10.1093/nargab/lqaa097 (2020). 10.1093/nargab/lqaa097 PMC7679065 33575641

[18] Wang, Y. J. & Kaestner, K. H. Single-cell RNA-Seq of the pancreatic islets—A promise not yet fulfilled?. *Cell Metab.* **29**, 539–544. 10.1016/j.cmet.2018.11.016 (2019). 30581120 10.1016/j.cmet.2018.11.016 PMC6402960

[19] Ngara, M. & Wierup, N. Lessons from single-cell RNA sequencing of human islets. *Diabetologia* **65**, 1241–1250. 10.1007/s00125-022-05699-1 (2022). 35482056 10.1007/s00125-022-05699-1 PMC9283180

[20] Denisenko, E. et al. Systematic assessment of tissue dissociation and storage biases in single-cell and single-nucleus RNA-seq workflows. *Genome Biol.* **21**, 130. 10.1186/s13059-020-02048-6 (2020). 32487174 10.1186/s13059-020-02048-6 PMC7265231

[21] Slyper, M. et al. A single-cell and single-nucleus RNA-Seq toolbox for fresh and frozen human tumors. *Nat. Med.* **26**, 792–802. 10.1038/s41591-020-0844-1 (2020). 32405060 10.1038/s41591-020-0844-1 PMC7220853

[22] Qadir, M. M. F. et al. Sex-specific regulatory architecture of pancreatic islets from subjects with and without type 2 diabetes. *EMBO J.* **43**, 6364–6382. 10.1038/s44318-024-00313-z (2024). 39567827 10.1038/s44318-024-00313-z PMC11649919

[23] Wang, G. et al. Integrating genetics with single-cell multiomic measurements across disease states identifies mechanisms of beta cell dysfunction in type 2 diabetes. *Nat. Genet.* **55**, 984–994. 10.1038/s41588-023-01397-9 (2023). 37231096 10.1038/s41588-023-01397-9 PMC10550816

[24] Basile, G. et al. Using single-nucleus RNA-sequencing to interrogate transcriptomic profiles of archived human pancreatic islets. *Genome Med.* **13**, 128. 10.1186/s13073-021-00941-8 (2021). 34376240 10.1186/s13073-021-00941-8 PMC8356387

[25] Kang, R. B. et al. Single-nucleus RNA sequencing of human pancreatic islets identifies novel gene sets and distinguishes beta-cell subpopulations with dynamic transcriptome profiles. *Genome Med.* **15**, 30. 10.1186/s13073-023-01179-2 (2023). 37127706 10.1186/s13073-023-01179-2 PMC10150516

[26] Xie, G. et al. NKX2-2 based nuclei sorting on frozen human archival pancreas enables the enrichment of islet endocrine populations for single-nucleus RNA sequencing. *BMC Genom.* **25**, 427. 10.1186/s12864-024-10335-w (2024). 10.1186/s12864-024-10335-w PMC11059690 38689254

[27] Baron, M. et al. A single-cell transcriptomic map of the human and mouse pancreas reveals inter- and intra-cell population structure. *Cell Syst.* **3**, 346-360.e344. 10.1016/j.cels.2016.08.011 (2016). 27667365 10.1016/j.cels.2016.08.011 PMC5228327

[28] Grun, D. et al. De Novo prediction of stem cell identity using single-cell transcriptome data. *Cell Stem Cell* **19**, 266–277. 10.1016/j.stem.2016.05.010 (2016). 27345837 10.1016/j.stem.2016.05.010 PMC4985539

[29] Muraro, M. J. et al. A single-cell transcriptome atlas of the human pancreas. *Cell Syst.* **3**, 385-394.e383. 10.1016/j.cels.2016.09.002 (2016). 27693023 10.1016/j.cels.2016.09.002 PMC5092539

[30] Elgamal, R. M. et al. An integrated map of cell type-specific gene expression in pancreatic islets. *Diabetes* **72**, 1719–1728. 10.2337/db23-0130 (2023). 37582230 10.2337/db23-0130 PMC10588282

[31] Bakken, T. E. et al. Single-nucleus and single-cell transcriptomes compared in matched cortical cell types. *PLoS ONE* **13**, e0209648. 10.1371/journal.pone.0209648 (2018). 30586455 10.1371/journal.pone.0209648 PMC6306246

[32] Wu, H., Kirita, Y., Donnelly, E. L. & Humphreys, B. D. Advantages of single-nucleus over single-cell RNA sequencing of adult kidney: Rare cell types and novel cell states revealed in fibrosis. *J. Am. Soc. Nephrol.* **30**, 23–32. 10.1681/ASN.2018090912 (2019). 30510133 10.1681/ASN.2018090912 PMC6317600

[33] Andrews, T. S. et al. Single-cell, single-nucleus, and spatial RNA sequencing of the human liver identifies cholangiocyte and mesenchymal heterogeneity. *Hepatol. Commun.* **6**, 821–840. 10.1002/hep4.1854 (2022). 34792289 10.1002/hep4.1854 PMC8948611

[34] Friberg, A. S. et al. Quantification of the islet product: Presentation of a standardized current good manufacturing practices compliant system with minimal variability. *Transplantation* **91**, 677–683. 10.1097/TP.0b013e31820ae48e (2011). 21248660 10.1097/TP.0b013e31820ae48e

[35] Young, M. D. & Behjati, S. SoupX removes ambient RNA contamination from droplet-based single-cell RNA sequencing data. *Gigascience.*10.1093/gigascience/giaa151 (2020). 33367645 10.1093/gigascience/giaa151 PMC7763177

[36] Hao, Y. et al. Integrated analysis of multimodal single-cell data. *Cell* **184**, 3573-3587.e3529. 10.1016/j.cell.2021.04.048 (2021). 34062119 10.1016/j.cell.2021.04.048 PMC8238499

[37] Germain, P. L., Lun, A., GarciaMeixide, C., Macnair, W. & Robinson, M. D. Doublet identification in single-cell sequencing data using scDblFinder. *F1000Res* **10**, 979. 10.12688/f1000research.73600.2 (2021). 35814628 10.12688/f1000research.73600.1 PMC9204188

[38] NBIS Workshop: Single Cell RNA-seq analysis. 2025 [Available from: https://nbisweden.github.io/workshop-scRNAseq/labs/seurat/seurat_01_qc.html#meta-qc_calqc]

[39] Brennecke, P. et al. Accounting for technical noise in single-cell RNA-seq experiments. *Nat. Methods* **10**, 1093–1095. 10.1038/nmeth.2645 (2013). 24056876 10.1038/nmeth.2645

[40] Hohmeier, H. E. et al. Isolation of INS-1-derived cell lines with robust ATP-sensitive K+ channel-dependent and -independent glucose-stimulated insulin secretion. *Diabetes* **49**, 424–430. 10.2337/diabetes.49.3.424 (2000). 10868964 10.2337/diabetes.49.3.424

[41] Lee MY, G. O., El-Mekkoussi H, Conery M, Manduchi E, Schug J,Descamps H, Lahori D, Da T, Liu C, Naji A, Voight BF, Li M, Kaestner KH. Single-cell multiome analysis supports α-to-β transdifferentiation in human pancreas. 10.1101/2025.02.14.638309

[42] Yamawaki, T. M. et al. Systematic comparison of high-throughput single-cell RNA-seq methods for immune cell profiling. *BMC Genom.* **22**, 66. 10.1186/s12864-020-07358-4 (2021). 10.1186/s12864-020-07358-4 PMC7818754 33472597

[43] Lehrstrand, J. et al. Illuminating the complete ss-cell mass of the human pancreas- signifying a new view on the islets of Langerhans. *Nat. Commun.* **15**, 3318. 10.1038/s41467-024-47686-7 (2024). 38632302 10.1038/s41467-024-47686-7 PMC11024155

[44] Murrall, K. *et al.* Small things matter: Lack of extra-islet beta cells in Type 1 diabetes. *bioRxiv* (2025). 10.1126/sciadv.adz2251 PMC12609120 41223263

[45] Itoh, N. & Okamoto, H. Translational control of proinsulin synthesis by glucose. *Nature* **283**, 100–102. 10.1038/283100a0 (1980). 6985712 10.1038/283100a0

[46] Stuart, T. et al. Comprehensive integration of single-cell data. *Cell* **177**, 1888-1902.e1821. 10.1016/j.cell.2019.05.031 (2019). 31178118 10.1016/j.cell.2019.05.031 PMC6687398

