# Supplementary material for: Evaluating cell-specific gene expression using single-cell and single-nuclei RNA-sequencing data from human pancreatic islets of the same donors
Source: Sci Rep. 2025 Oct 16;15:36133. doi: 10.1038/s41598-025-21595-1 (PMC12533216; doi:10.1038/s41598-025-21595-1)
Supplement: Supplementary file 1 — Supplementary Information 1. [file 41598_2025_21595_MOESM1_ESM.pdf]

Evaluating cell-specific gene expression using single-cell and single-nuclei RNA-sequencing data from human pancreatic islets of the same donors

Karin Engström[a]*, Åsa Nilsson[b]*, Jones K. Ofori[c], Nils Wierup[d], Karl Bacos[c], and Charlotte Ling[c]

*shared

[a] Epidemiology and Bioinformatics, Division of Occupational and Environmental Medicine, Department of Laboratory Medicine, Lund University, Sweden

[b] Human Tissue Lab, Department of Clinical Sciences in Malmö, Lund University Diabetes Centre, Lund University, Scania University Hospital, Malmö, Sweden

[c] Epigenetics and Diabetes Unit, Department of Clinical Sciences in Malmö, Lund University Diabetes Centre, Lund University, Scania University Hospital, Malmö, Sweden

[d] Neuroendocrine Cell Biology, Department of Experimental Medical Science, Lund University Diabetes Centre, Lund University, Scania University Hospital, Malmö, Sweden

Corresponding authors: Karin Engström, karin.engstrom@med.lu.se, and Charlotte Ling, charlotte.ling@med.lu.se

Supplementary Figure 1. Violin plots depicting quality control (QC) metrics per donor, stratified by modality (scRNA-seq [cells] vs. snRNA-seq [nuclei]). Each violin shows the full distribution of cells for a given donor and modality.

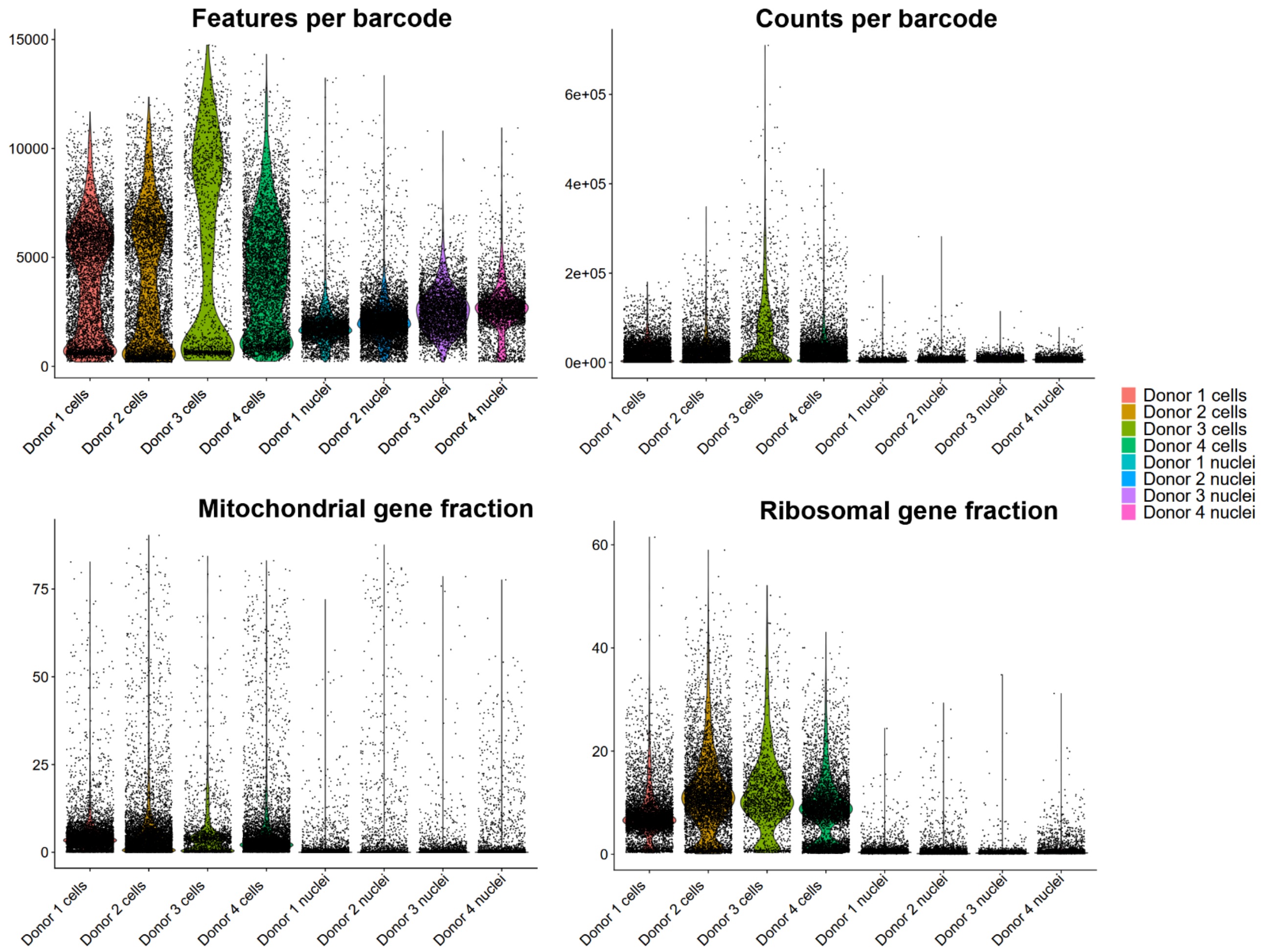

Supplementary Figure 2. The upper figure displays a UMAP of the barcodes labelled by cells or nuclei, while the lower figure shows the number of barcodes per cluster labelled by cells or nuclei.

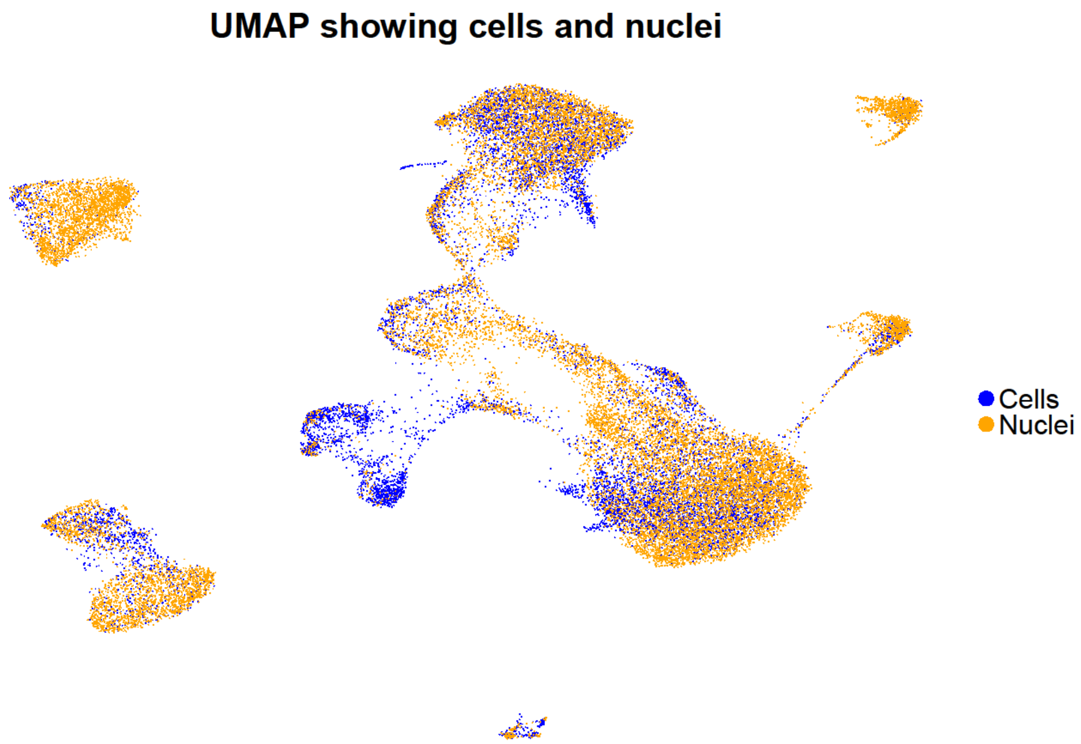

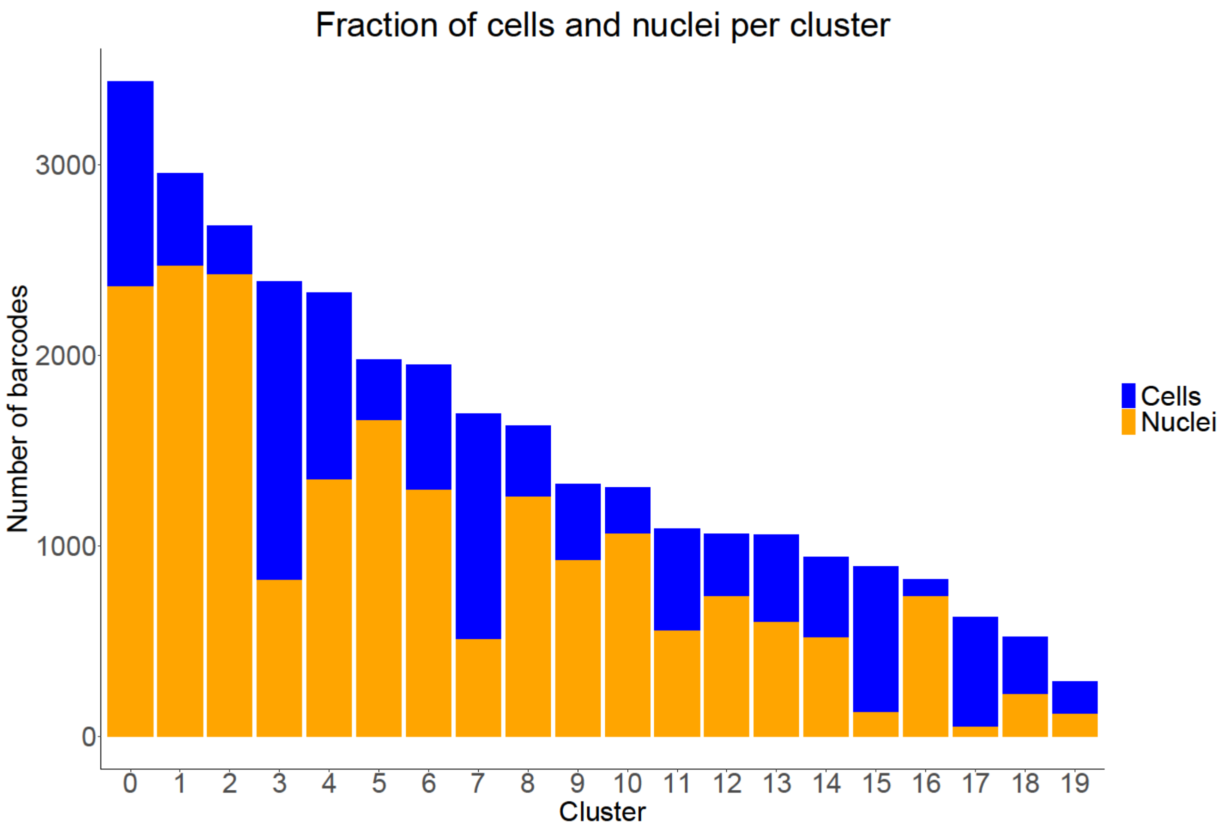

Supplementary Figure 3. (a–c) shows predicted cell type proportions per donor, stratified by modality (scRNA-seq and snRNA-seq) and shown for the three annotation methods (Manual, Azimuth, HPAP). Each bar represents the relative proportion of annotated cell types within an individual donor and modality (n = 4 donors). (d) Paired differences in predicted cell type proportions (snRNA-seq – scRNA-seq) per cell type and annotation method. Points indicate the Hodges–Lehmann (HL) estimate of the median paired shift across the four donors, with error bars showing 95% confidence intervals from paired Wilcoxon signed-rank tests. Statistical significance was determined using paired Wilcoxon signed-rank tests with Benjamini–Hochberg correction for multiple testing. (e) Weighted Jaccard indices quantifying the overlap of predicted cell types between scRNA-seq and snRNA-seq across the four donors, per annotation method. Boxplots show the interquartile range (IQR), the horizontal line denotes the median, and whiskers extend to 1.5× the IQR.

FIGURE ON NEXT PAGE

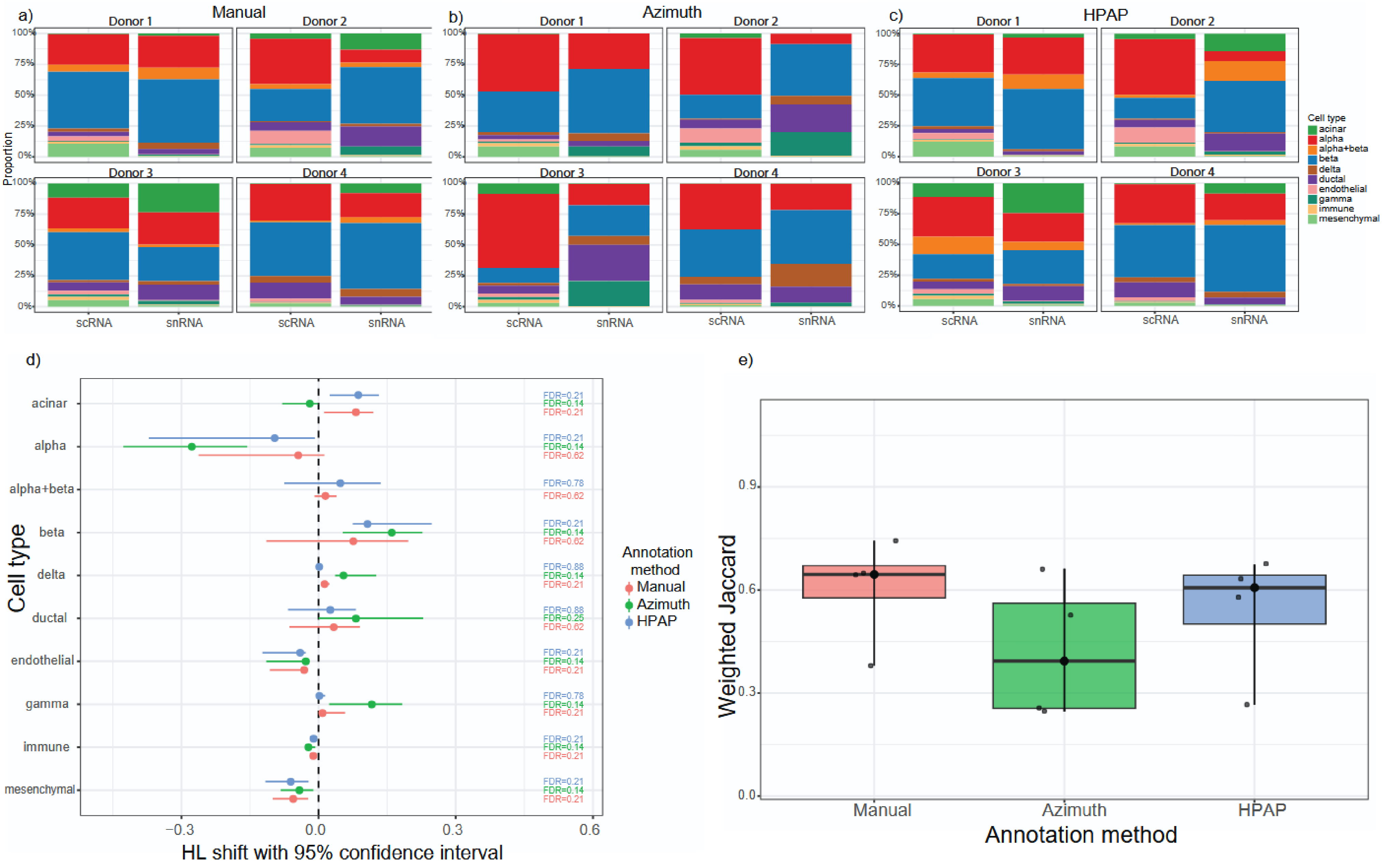

a)
Manual
b)
Azimuth
c)
HPAP
Donor 1
Donor 2
Donor 3
Donor 4
Proportion
100%
75%
50%
25%
0%
scRNA
snRNA
Cell type
acinar
alpha
alpha+beta
beta
delta
ductal
endothelial
gamma
immune
mesenchymal
d)
acinar
alpha
alpha+beta
beta
delta
ductal
endothelial
gamma
immune
mesenchymal
Cell type
-0.3
0.0
0.3
0.6
HL shift with 95% confidence interval
Annotation method
Manual
Azimuth
HPAP
e)
Weighted Jaccard
0.0
0.3
0.6
0.9
Manual
Azimuth
HPAP
Annotation method

Supplementary Figure 4. Barplots comparing the annotated cell types from two different annotation methods.

Supplementary Figure 4 is shown as a separate file since it is too large to fit into Document S1.

Supplementary Figure 5. Venn diagram showing the overlap between scRNA-seq and snRNA-seq for all genes detected (upper) and genes expressed (defined as UMI≥3) in at least 10% of the cells/nuclei (lower) in islets from four donors. The upper panel depicts the overlap of all genes detected, while the lower panel shows genes expressed in at least 10% of cells or nuclei. Gene detection was summarized across all donors for each modality.

Overlap scRNAseq and snRNAseq, all detected genes

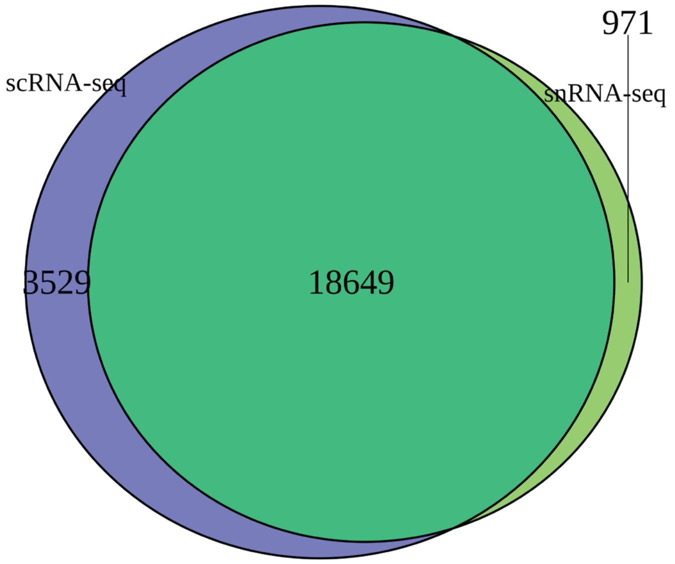

Overlap scRNAseq and snRNAseq, genes expressed at 10%

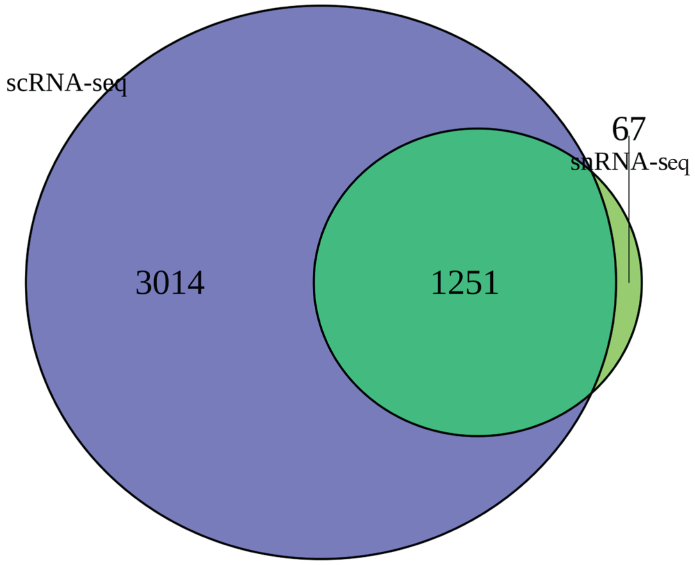

Supplementary Figure 6. UMAP showing manually annotated cell types, including the stressed beta cell type. The figure is split by scRNA-seq (left) and snRNA-seq (right).

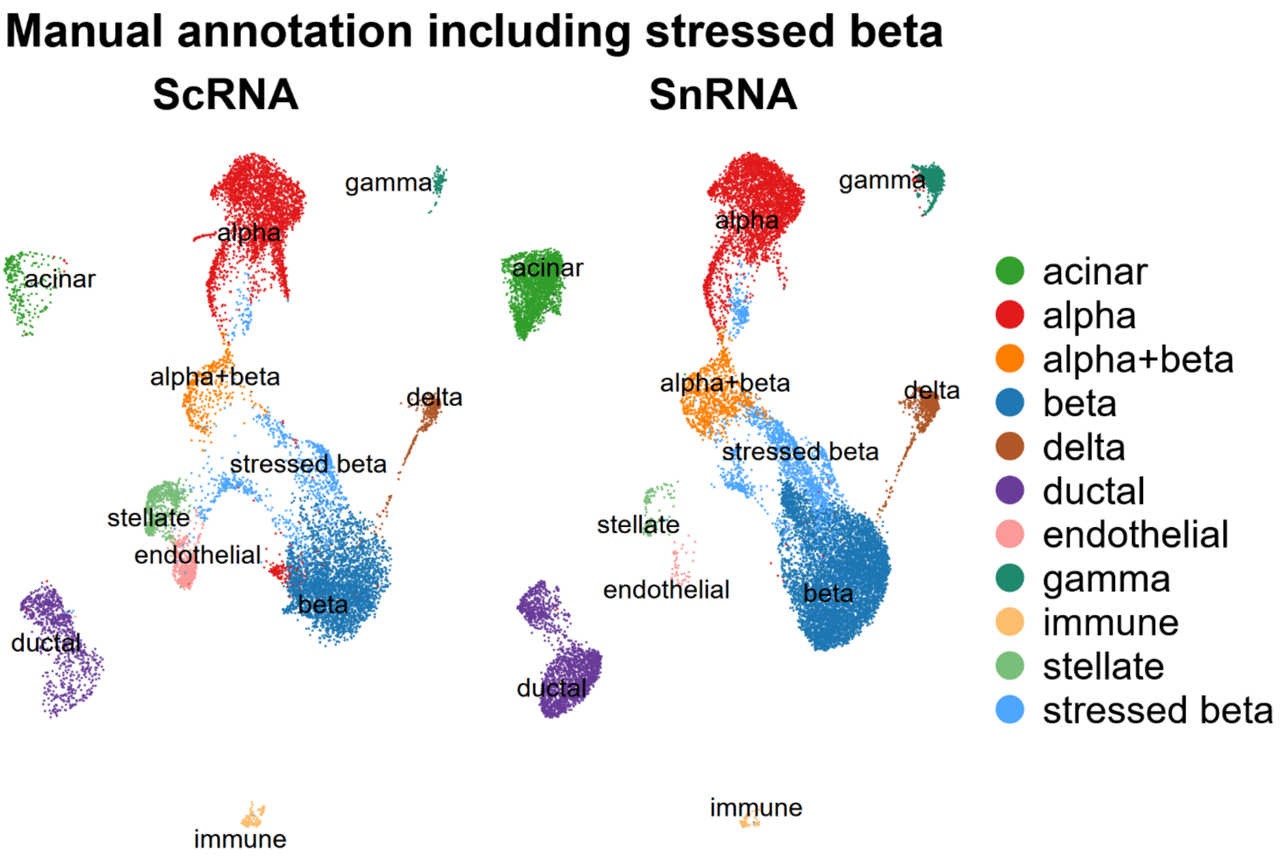

Supplementary Figure 7. (a) Slope plots of per-donor marker gene detection rates. The fraction of cells or nuclei expressing the indicated marker genes (UMI ≥ 3) in their respective target cell type is shown, stratified by donor and modality. Each line connects paired scRNA-seq (left) and snRNA-seq (right) samples from the same donor, illustrating within-donor differences. Points represent detection proportions per donor and modality. (b) Paired Wilcoxon signed-rank test of marker gene detection rates. For each marker gene and target cell type, paired differences in detection rate (snRNA–scRNA) across the four donors are summarized. Each point shows the median paired difference, with error bars indicating the 95% confidence interval of the Hodges–Lehmann shift estimate. A dashed horizontal line at zero indicates no difference between modalities.

FIGURE ON NEXT PAGE

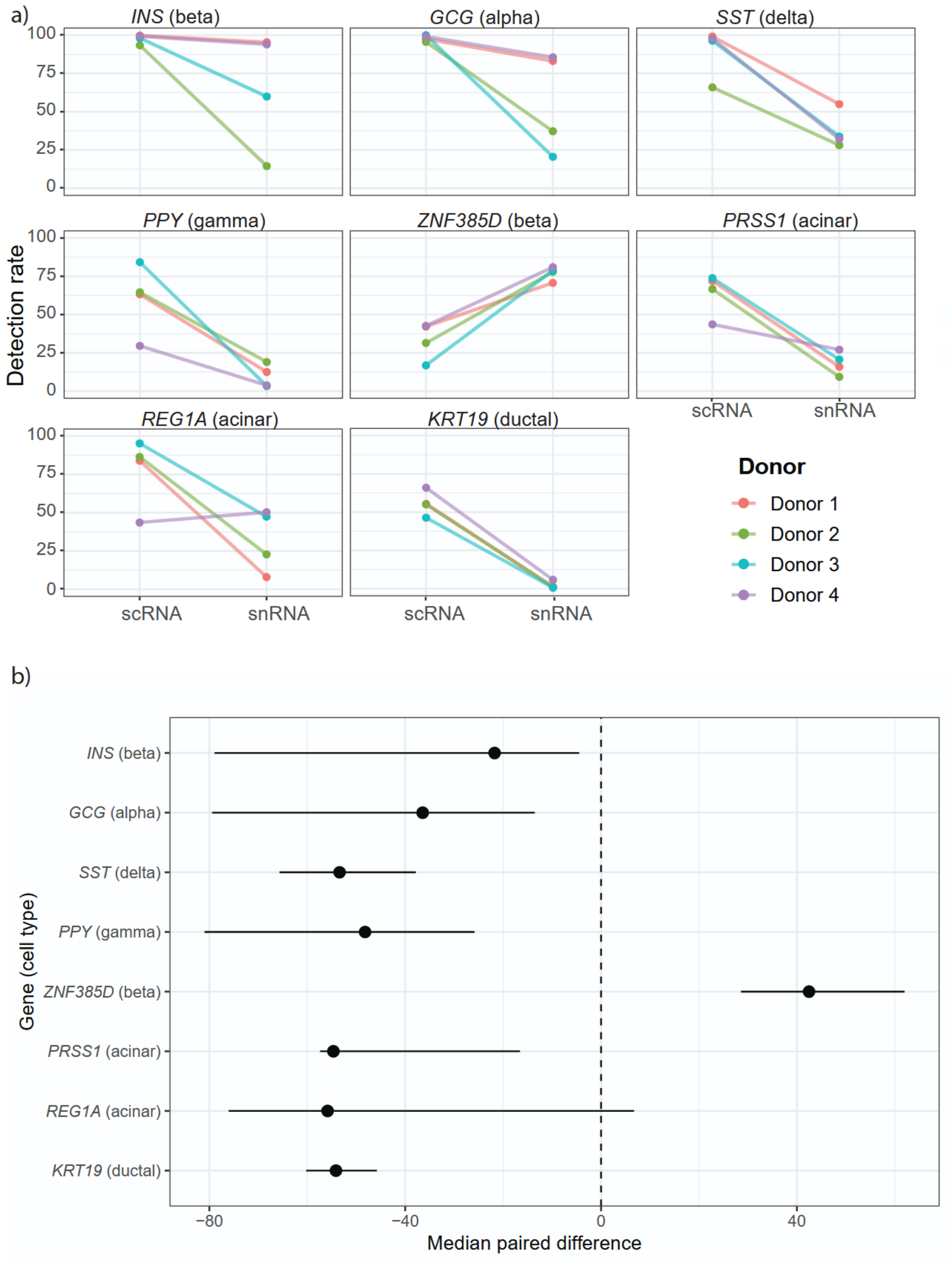
a)
INS (beta)
GCG (alpha)
SST (delta)
PPY (gamma)
ZNF385D (beta)
PRSS1 (acinar)
REG1A (acinar)
KRT19 (ductal)
Detection rate
100
75
50
25
0
scRNA
snRNA
Donor
Donor 1
Donor 2
Donor 3
Donor 4
b)
INS (beta)
GCG (alpha)
SST (delta)
PPY (gamma)
ZNF385D (beta)
PRSS1 (acinar)
REG1A (acinar)
KRT19 (ductal)
Gene (cell type)
−80
−40
0
40
Median paired difference

Supplementary Figure 8. Violin plots showing expression distributions of selected canonical and snRNA-seq marker genes in their respective cell types in scRNA-seq (red) and snRNA-seq (blue). Each violin represents the distribution of log-normalized expression values, with dots indicating individual observations. Statistical comparisons per cell type between modalities were performed using paired Wilcoxon signed-rank tests at the donor level, with 95% confidence intervals for the Hodges–Lehmann shift.

FIGURE ON NEXT PAGE

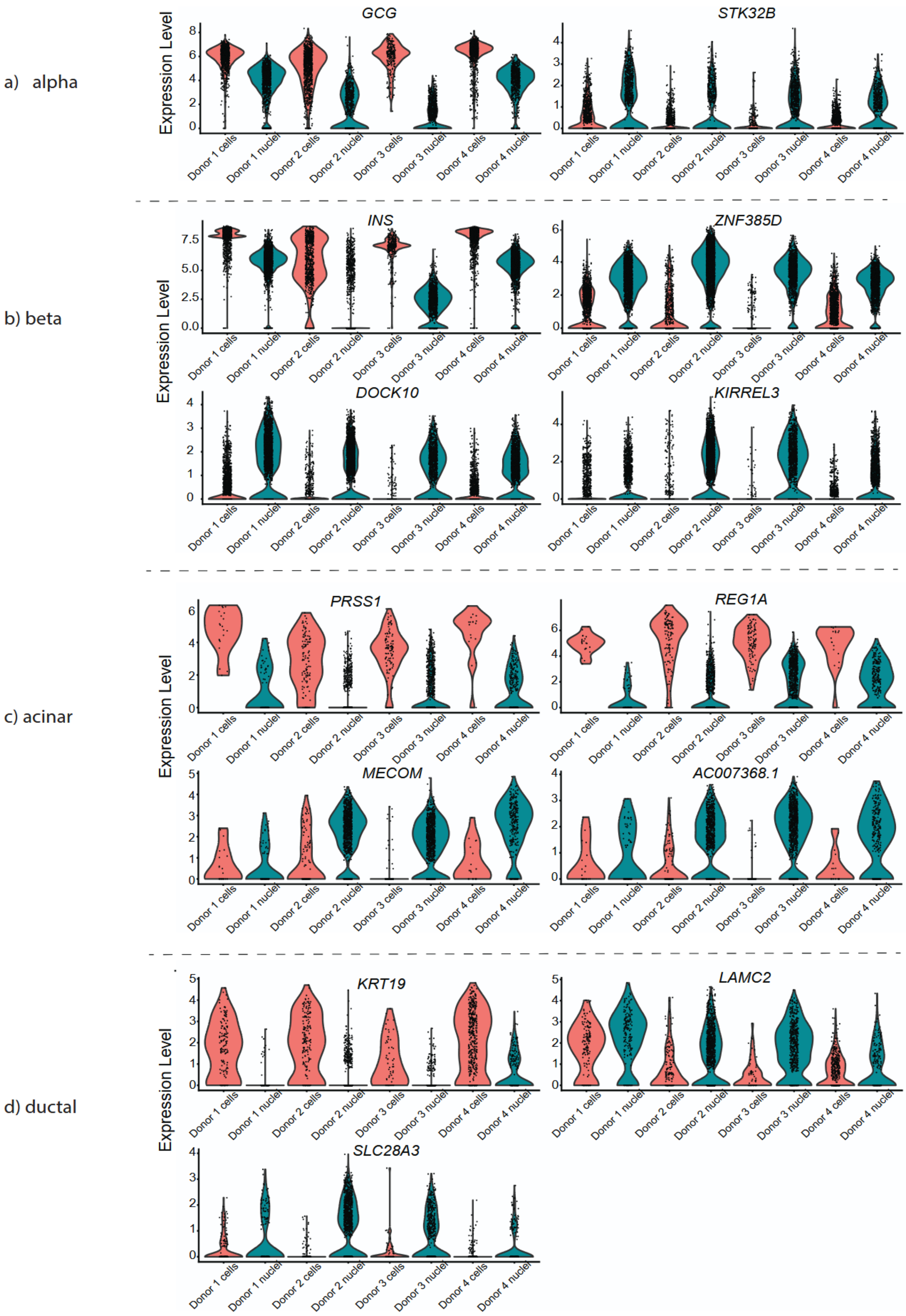

a) alpha
b) beta
c) acinar
d) ductal
Expression Level
GCG
STK32B
INS
ZNF385D
DOCK10
KIRREL3
PRSS1
REG1A
MECOM
AC007368.1
KRT19
LAMC2
SLC28A3
Donor 1 cells
Donor 1 nuclei
Donor 2 cells
Donor 2 nuclei
Donor 3 cells
Donor 3 nuclei
Donor 4 cells
Donor 4 nuclei

Supplementary Figure 9. Dot plots showing the expression of selected canonical and snRNA-seq marker genes across predicted cell types in snRNA-seq, annotated using Manual, Azimuth, and HPAP methods. Only cell types present in all three annotation methods are shown, e.g., the category alpha+beta cells, not present in Azimth annotation, is omitted. Dot size indicates the percentage of cells expressing a given gene within each cell type, while color intensity represents average expression level. Dashed lines separate marker genes by target cell type, with marker genes ordered to correspond to their expected cell type in the same order as presented on the y-axis (e.g., alpha cell markers on the left). Plots were generated with Seurat's DotPlot function and are descriptive.

FIGURE ON NEXT PAGE

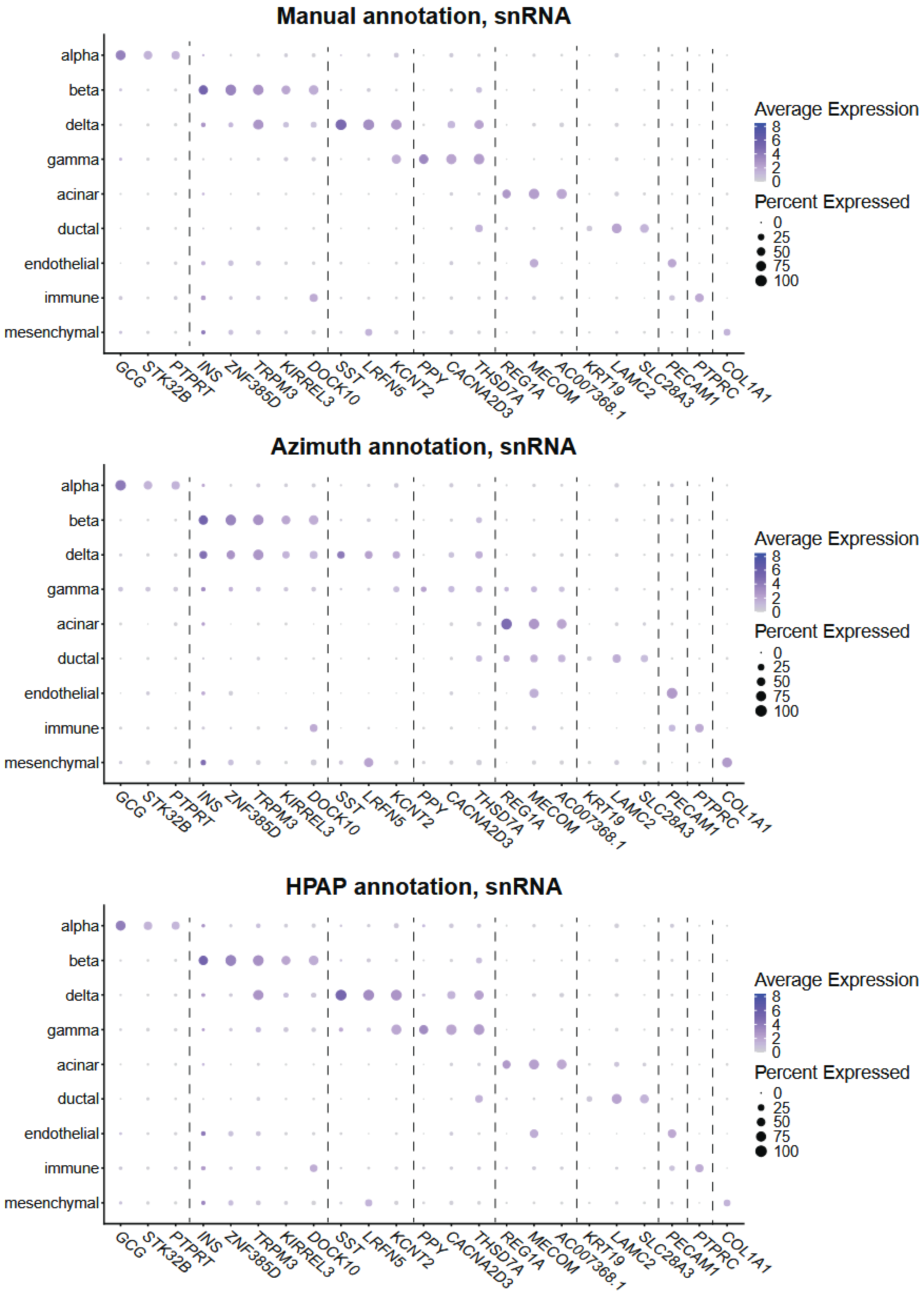
Manual annotation, snRNA
alpha
beta
delta
gamma
acinar
ductal
endothelial
immune
mesenchymal
GCG
STK32B
PTPRT
INS
ZNF385D
TRPM3
KIRREL3
DOCK10
SST
LRFN5
KCNT2
PPY
CACNA2D3
THSD7A
REG1A
MECOM
AC007368.1
KRT19
LAMC2
SLC28A3
PECAM1
PTPRC
COL1A1
Average Expression
8
6
4
2
0
Percent Expressed
0
25
50
75
100
Azimuth annotation, snRNA
alpha
beta
delta
gamma
acinar
ductal
endothelial
immune
mesenchymal
GCG
STK32B
PTPRT
INS
ZNF385D
TRPM3
KIRREL3
DOCK10
SST
LRFN5
KCNT2
PPY
CACNA2D3
THSD7A
REG1A
MECOM
AC007368.1
KRT19
LAMC2
SLC28A3
PECAM1
PTPRC
COL1A1
Average Expression
8
6
4
2
0
Percent Expressed
0
25
50
75
100
HPAP annotation, snRNA
alpha
beta
delta
gamma
acinar
ductal
endothelial
immune
mesenchymal
GCG
STK32B
PTPRT
INS
ZNF385D
TRPM3
KIRREL3
DOCK10
SST
LRFN5
KCNT2
PPY
CACNA2D3
THSD7A
REG1A
MECOM
AC007368.1
KRT19
LAMC2
SLC28A3
PECAM1
PTPRC
COL1A1
Average Expression
8
6
4
2
0
Percent Expressed
0
25
50
75
100

Supplementary Figure 10. UMAP showing the expression of *INS*, *GCG*, and co-expression of *INS* and *GCG* in a) scRNA-seq and b) snRNA-seq. Cells/nuclei with a high expression of both *INS* and *GCG* are colored yellow in the plot depicting the co-expression. Two nuclei are excluded from the snRNA-seq figure due to y-axis values of 8 and 10 to avoid making the cluster visualization too small; both nuclei had low INS and GCG expression (grey dots). The color scheme of the co-expression plots is shown in the upper right part of the figure, where the expression values on the x and y axes are log-normalized. Panel c) shows the expression of *TPH2*, a marker gene for the alpha+beta cluster, in scRNA-seq and snRNA-seq.

FIGURE ON NEXT PAGE

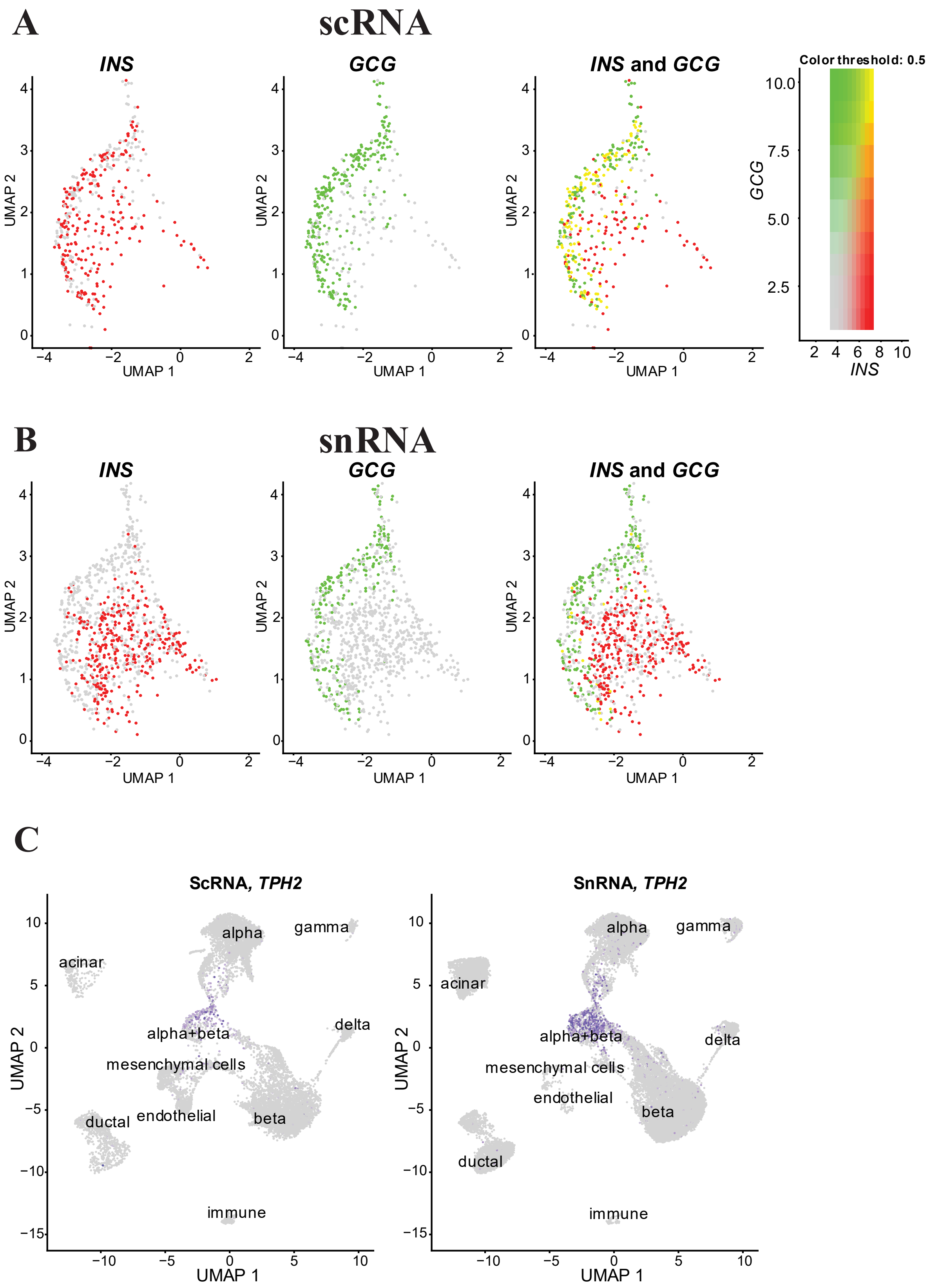
A
scRNA
INS
GCG
INS and GCG
Color threshold: 0.5
GCG
INS
UMAP 1
UMAP 2
B
snRNA
INS
GCG
INS and GCG
UMAP 1
UMAP 2
C
ScRNA, TPH2
SnRNA, TPH2
alpha
gamma
acinar
alpha+beta
delta
mesenchymal cells
endothelial
ductal
beta
immune
UMAP 1
UMAP 2

Supplementary Table 1. Marker genes identified by the FindAllMarkers analysis and used for manual cluster annotation. The table lists marker genes per cluster, along with associated statistics (p-value, average log_2 fold change, the percentage of cells where the gene is detected (UMI≥ 1) in the cell type of interest, the percentage of cells where the feature is detected in the second group (average across all other genes, not for a specific cluster), and adjusted p-value (FDR).

Table, too large to fit into Document S1 and submitted separately

Supplementary Table 2. Paired Wilcoxon signed-rank test comparing cell-type proportions between annotation methods within the same modality (scRNA-seq or snRNA-seq). Each row represents a contrast between two annotation methods (e.g., "azimuth – manual" indicates Azimuth compared to Manual). Reported values include raw p-values (p_value) and adjusted p-values (Benjamini–Hochberg, padj_BH), Hodges–Lehmann estimate of the median paired difference (HL_shift), 95% confidence intervals (CI_low, CI_high), and median difference in proportions (median_diff).

Table, too large to fit into Document S1 and submitted separately

upplementary Table 3. Prediction scores for Azimuth and HPAP annotations split for scRNA-seq and snRNA-seq data from human pancreatic islets of four donors. Prediction scores assess the confidence in the cell type labels assigned to each barcode based on similarity to the reference.

Cell type	Azimuth median, scRNA	Azimuth Q1, scRNA	Azimuth Q3, scRNA	Azimuth median, snRNA	Azimuth Q1, snRNA	Azimuth Q3, snRNA	HPAP median, scRNA	HPAP Q1, scRNA	HPAP Q3, scRNA	HPAP median, snRNA	HPAP Q1, snRNA	HPAP Q3, snRNA
**Acinar**	1	0.99	1	0.45	0.38	0.57	1	0.95	1	0.91	0.71	1
**Activated_stellate**	0.70	0.58	0.90	0.79	0.57	0.93	1	0.94	1	0.97	0.79	1
**Alpha**	1	1	1	0.79	0.53	0.99	1	0.98	1	0.84	0.68	0.92
**Alpha+Beta**							0.64	0.54	0.79	0.66	0.55	0.79
**Beta**	1	0.95	1	0.66	0.53	0.80	1	0.90	1	0.96	0.86	1.00
**Cycling**	0.78	0.75	0.81									
**Cycling Alpha**							0.77	0.55	0.98	0.97	0.92	0.99
**Delta**	0.99	0.78	1	0.45	0.40	0.61	0.83	0.65	0.92	0.65	0.52	0.78
**Ductal**	1	1.00	1	0.94	0.77	1.00	0.87	0.68	1	0.99	0.89	1
**MUC5B+ Ductal**							0.69	0.58	0.83	0.66	0.52	0.77
**Endothelial**	1	0.99	1	0.89	0.63	0.99	1	0.99	1	1	0.97	1
**Gamma**	0.75	0.45	0.95	0.46	0.40	0.60						
**Gamma+Epsilon**							0.65	0.50	0.82	0.54	0.45	0.63
**Immune**	0.92	0.50	0.97	0.83	0.58	0.96						
**Macrophage**							1	0.92	1	0.83	0.76	0.87
**Mast**							0.94	0.88	0.98	0.77	0.47	1
**Quiescent_stellate**	0.63	0.52	0.81	0.71	0.68	0.80	0.96	0.78	0.99	0.97	0.95	0.98
**Schwann**	0.39	0.32	0.97	0.24	0.22	0.26						
**All cell types**	1	0.96	1	0.67	0.48	0.88	1	0.88	1	0.90	0.73	0.98

Supplementary Table 4. Mapping scores for Azimuth split for scRNA-seq and snRNA-seq data from human pancreatic islets of four donors.

Mapping scores assess the confidence in how well a query barcode's overall transcriptome aligns with the reference embedding.

Cell type	Azimuth median, scRNA	Azimuth Q1, scRNA	Azimuth Q3, scRNA	Azimuth median, snRNA	Azimuth Q1, snRNA	Azimuth Q3, snRNA
**Acinar**	0.97	0.94	0.99	0.22	0.14	0.34
**Activated_stellate**	0.73	0.67	0.84	0.59	0.49	0.76
**Alpha**	0.76	0.62	0.86	0.33	0.25	0.42
**Beta**	0.81	0.71	0.89	0.15	0.11	0.25
**Cycling**	0.95	0.95	0.96			
**Delta**	0.79	0.51	0.94	0.34	0.22	0.45
**Ductal**	0.88	0.72	0.94	0.37	0.28	0.54
**Endothelial**	0.85	0.74	0.91	0.70	0.56	0.76
**Gamma**	0.63	0.43	0.87	0.36	0.23	0.48
**Immune**	0.95	0.64	0.99	0.33	0.27	0.44
**Quiescent_stellate**	0.70	0.60	0.82	0.51	0.41	0.71
**Schwann**	0.72	0.63	0.95	0.41	0.37	0.49
**All cell types**	0.79	0.66	0.89	0.27	0.16	0.40

Supplementary Table 5. Genes expressed per cell type at a detection level of 3 UMIs. The fraction of barcodes per modality for which the genes are expressed is shown.

Too large to fit into Document S1 and submitted seperately.

Supplementary Table 6. Number of detected genes (UMI ≥ 3) per manually annotated cell type for snRNA-seq and scRNA-seq, as well as the overlap between detected features for scRNA-seq and snRNA-seq data from human pancreatic islets of four donors.

	All detected genes						Genes detected at a frequency above 10%					
**Annotated cell type**	**scRNA-seq**	**snRNA-seq**	**scRNA-seq, unique genes**	**snRNA-seq, unique genes**	**Overlap scRNA-seq and snRNA-seq**	**Overlap scRNA-seq genes and snRNA-seq genes, %**	**scRNA-seq**	**snRNA-seq**	**scRNA-seq, unique genes**	**snRNA-seq, unique genes**	**Overlap scRNA-seq and snRNA-seq**	**Overlap snRNA-seq genes and scRNA-seq genes %**
Acinar	11069	12518	1595	3044	9474	76	2447	1127	1502	182	945	39
Alpha	17137	14265	3743	871	13394	78	5050	1332	3730	12	1320	26
Alpha+beta	15635	12643	4248	1256	11387	73	1871	538	1385	52	486	26
Beta	17096	16235	2716	1855	14380	84	3088	1382	1961	255	1127	36
Delta	13101	12139	2647	1685	10454	80	4199	1552	2711	64	1488	35
Ductal	15949	12933	3850	834	12099	76	5181	1356	3848	23	1333	26
Endothelial	13051	5275	8020	244	5031	38	4389	1243	3200	54	1189	27
Gamma	11915	10046	3140	1271	8775	74	5675	1219	4459	3	1216	21
Immune	10722	6395	5011	684	5711	53	2533	953	1666	86	867	34
Stellate	15983	8685	7499	201	8484	53	5023	1522	3541	40	1482	29

Supplementary Table 7. List of marker genes in the findAllMarkers analysis performed to evaluate marker genes for the annotated cell types, with one sheet for scRNA-seq and one sheet for snRNA-seq. The table lists marker genes per cell type, along with associated statistics (p-value, average $\log_2$ fold change, the percentage of cells where the gene is detected (UMI$\geq$ 1) in the cell type of interest, the percentage of cells where the feature is detected in the second group (average across all other genes, not for a specific cell type), and adjusted p-value (FDR).

Too large to fit into Document S1 and submitted seperately.

Supplementary Table 8. List of marker genes for snRNA-seq that overlapped with the marker genes from the study of Kang et al[a] for selected cell types, presented in one sheet per cell type.

Description of the data: Table, too large to fit into Document S1.

[a] Kang RB, Li Y, Rosselot C, Zhang T, Siddiq M, Rajbhandari P, et al. Single-nucleus RNA sequencing of human pancreatic islets identifies novel gene sets and distinguishes beta-cell subpopulations with dynamic transcriptome profiles. Genome Med. 2023;15(1):30)

Too large to fit into Document S1 and submitted seperately.

Supplementary Table 9. Results from FindAllMarkers analysis comparing scRNA-seq and snRNA-seq within each major cell type. Each sheet corresponds to one cell type and lists genes more highly expressed in scRNA-seq compared to snRNA-seq. Reported statistics include: p-value, average $\log_2$ fold change, percentage of cells with expression (UMI $\geq$ 1) in scRNA-seq, percentage of nuclei with expression in snRNA-seq, and adjusted p-value (FDR).

Too large to fit into Document S1 and submitted seperately.

Supplementary Table 10. Results from FindAllMarkers analysis comparing snRNA-seq and scRNA-seq within each major cell type. Each sheet corresponds to one cell type and lists genes more highly expressed in snRNA-seq compared to scRNA-seq. Reported statistics include: p-value, average $\log_2$ fold change, percentage of nuclei with expression (UMI $\geq$ 1) in snRNA-seq, percentage of cells with expression in scRNA-seq, and adjusted p-value (FDR).

Too large to fit into Document S1 and submitted seperately.
