# Supplementary figures and images for: Evaluating cell-specific gene expression using single-cell and single-nuclei RNA-sequencing data from human pancreatic islets of the same donors
Source: Sci Rep. 2025 Oct 16;15:36133. doi: 10.1038/s41598-025-21595-1 (PMC12533216; doi:10.1038/s41598-025-21595-1)

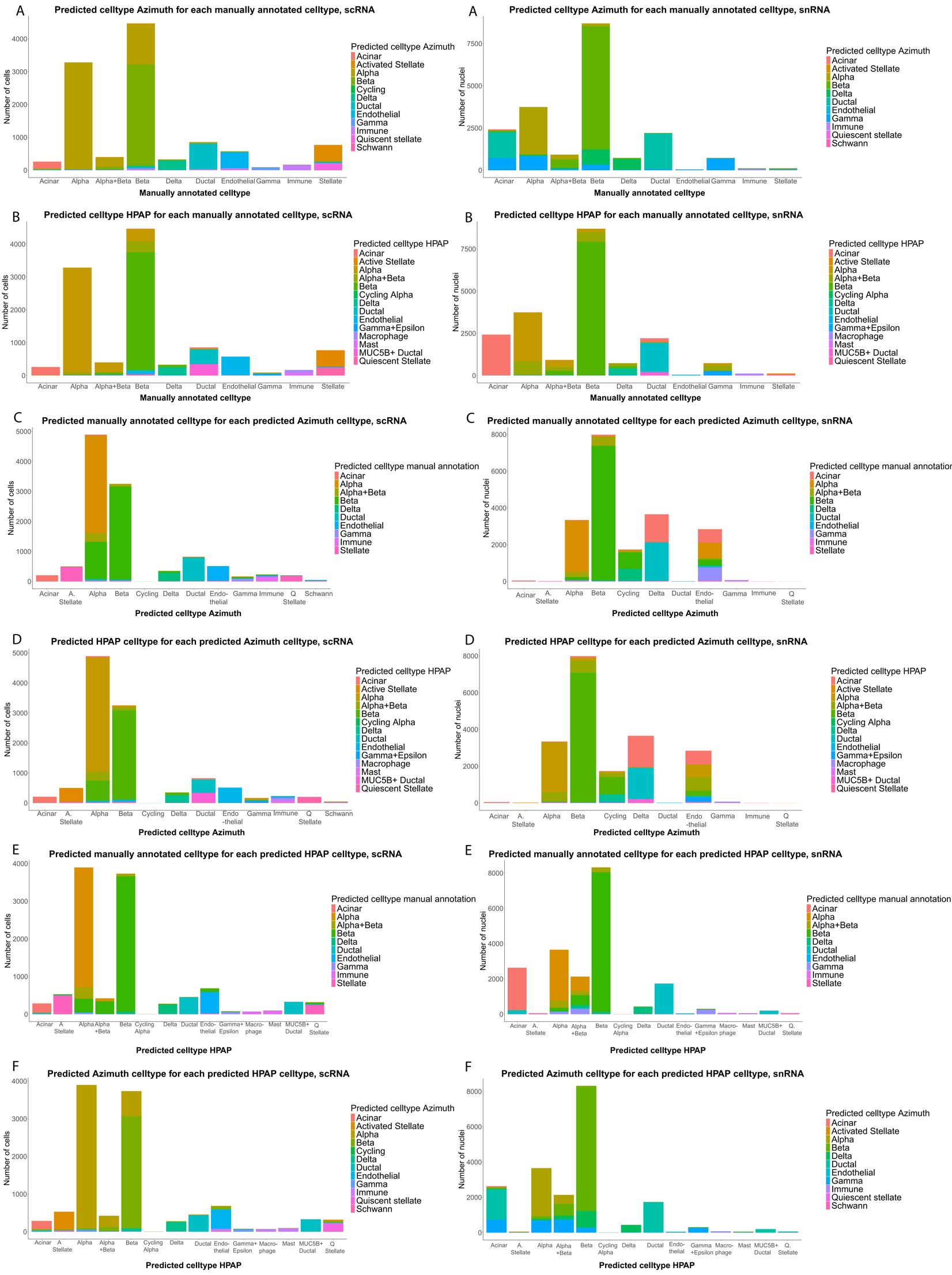

Supplement: Supplementary file 2 — Supplementary Information 2. [file 41598_2025_21595_MOESM2_ESM.pdf]
